# Phenotypic and molecular characterization of sweet sorghum accessions for bioenergy production

**DOI:** 10.1371/journal.pone.0183504

**Published:** 2017-08-17

**Authors:** Michele Jorge da Silva, Maria Marta Pastina, Vander Fillipe de Souza, Robert Eugene Schaffert, Pedro Crescêncio Souza Carneiro, Roberto Willians Noda, José Eustáquio de Souza Carneiro, Cynthia Maria Borges Damasceno, Rafael Augusto da Costa Parrella

**Affiliations:** 1 Departamento de Biologia Geral, Universidade Federal de Viçosa, Viçosa, Minas Gerais, Brasil; 2 Embrapa Milho e Sorgo, Sete Lagoas, Minas Gerais, Brasil; 3 Departamento de Engenharia de Biossistemas, Universidade Federal de São João del-Rei, São João del-Rei, Minas Gerais, Brasil; 4 Departamento de Fitotecnia, Universidade Federal de Viçosa, Viçosa, Minas Gerais, Brasil; CIRAD, FRANCE

## Abstract

Sweet sorghum [*Sorghum bicolor* (L.) Moench] is a type of cultivated sorghum characterized by the accumulation of high levels of sugar in the stems and high biomass accumulation, making this crop an important feedstock for bioenergy production. Sweet sorghum breeding programs that focus on bioenergy have two main goals: to improve quantity and quality of sugars in the juicy stem and to increase fresh biomass productivity. Genetic diversity studies are very important for the success of a breeding program, especially in the early stages, where understanding the genetic relationship between accessions is essential to identify superior parents for the development of improved breeding lines. The objectives of this study were: to perform phenotypic and molecular characterization of 100 sweet sorghum accessions from the germplasm bank of the Embrapa Maize and Sorghum breeding program; to examine the relationship between the phenotypic and the molecular diversity matrices; and to infer about the population structure in the sweet sorghum accessions. Morphological and agro-industrial traits related to sugar and biomass production were used for phenotypic characterization, and single nucleotide polymorphisms (SNPs) were used for molecular diversity analysis. Both phenotypic and molecular characterizations revealed the existence of considerable genetic diversity among the 100 sweet sorghum accessions. The correlation between the phenotypic and the molecular diversity matrices was low (0.35), which is in agreement with the inconsistencies observed between the clusters formed by the phenotypic and the molecular diversity analyses. Furthermore, the clusters obtained by the molecular diversity analysis were more consistent with the genealogy and the historic background of the sweet sorghum accessions than the clusters obtained through the phenotypic diversity analysis. The low correlation observed between the molecular and the phenotypic diversity matrices highlights the complementarity between the molecular and the phenotypic characterization to assist a breeding program.

## Introduction

The current policy in several countries, including Brazil, is to promote research and development on renewable energy sources [1–3]. Many countries are pursuing to increase the participation of biofuels in its energy mix and consequently to reduce carbon dioxide emissions in the atmosphere by decreasing the burning of fossil fuels [4]. In Brazil, sugarcane stands out as a feedstock for ethanol production [1,5], but the country has difficulty to meet its domestic demand, especially in the sugarcane off-season. Sweet sorghum [*Sorghum bicolor* (L.) Moench] is a type of domesticated sorghum characterized by the accumulation of high levels of sugar in the stems and high biomass production, making this crop an important alternative for bioethanol production, and cogeneration of energy [3,6–8]. Sorghum is a grass of African origin that resembles sugarcane, a close relative. Thus, sorghum juice can be easily extracted to produce ethanol in the same distilleries that process sugarcane. In addition, the sorghum harvest can be carried out during the sugarcane off-season just prior to the beginning of sugarcane processing, benefiting the ethanol industry. Besides these advantages, sweet sorghum cultivars that are insensitive to photoperiod have a vegetative cycle ranging from 90 to 130 days, much shorter than that of sugarcane [9–12].

Sweet sorghum accessions were introduced into the United States, from China and Africa, 150 years ago. The variety Chinese Amber was the first introduction of sweet sorghum into the USA, in 1853. Several cultivars came from Africa, such as Orange, Sumac, Redtop, Gooseneck, Texas Seed Cane Ribbon, Honey and White African [13]. The center of sorghum domestication is in central Africa, and the highest levels of genetic and phenotypic diversity in both cultivated and wild sorghum are found in this region [14]. Other cultivars were introduced over the years, such as Collier, South Africa, Mclean, Australia, and others of unknown origin, such as Folger, Coleman, Sugar Drip, and Rex [15,16]. Most modern sweet sorghum varieties were developed in the period 1940–1983 with support from the United States Department of Agriculture (USDA) and the Sugar Crops Field Station, located in city of Meridian, Mississippi. Landraces, i.e. inbred lines considered native to Africa, were used in several studies for the genetic improvement of sweet sorghum. In the 1850s, the main goal was to use sweet sorghum for the production of syrup, which reached about 136 million liters in 1946, replacing crystal sugar during World War II [17,18]. The focus was to develop materials for syrup production with disease resistance, high soluble solids content (Brix), good purity (high sucrose) and quality of sugars in the juice. The landraces MN 960, MN 1048, MN 1054, MN 1056, MN 1060 and MN 1500 were widely used in the early breeding programs in the United States [19].

Sorghum was introduced into Brazil in the early twentieth century, mostly through initiatives and efforts of research institutes and universities [20,21]. In 1976, influenced by the National Alcohol Program (in portuguese, Programa Nacional do Álcool—Pro-Álcool), Embrapa Maize and Sorghum initiated a research program for sweet sorghum cultivar development and feasibility studies for ethanol production, especially for use in small distilleries [20] to supply liquid fuel for agriculture expansion in the Central-west region of Brazil. However, the sweet sorghum breeding program was put on hold in the mid 1980′s with a modified government policy that focused the incentives only for large distilleries. Embrapa′s sweet sorghum breeding program was reactivated in 2008 following the guidelines of the Brazilian National Agro-Energy Plan (PNA 2006–2011) [22]. For the production of bioenergy, the main objectives of a sweet sorghum breeding program are to improve the quantity and quality of sugars in the extracted juicy from the stems and to increase green biomass productivity. A high-potential sweet sorghum cultivar should have the following features: high biomass yield capacity, lodging resistance, high percentage of extractable juice, high content of soluble solids in the juice, high purity of sugars, resistance to major diseases and tolerance to drought and waterlogging [23].

Sorghum is a species that exhibits a diverse set of agronomic and morphological characteristics [24,25]. Harlan and de Wet [26] classified sorghum into five major races: *bicolor*, *caudatum*, *durra*, *guinea* and *kafir* and 10 other hybrid races which are combinations of the basic races. This classification is simple and primarily based on morphological features of panicle and grain. However, sweet sorghum has not been bred for panicle or grain characteristics, and there are few insights about its origin. Therefore, the relationship between sweet sorghum and the traditional classification of major sorghum races is inconsistent [19][27]. Sweet sorghum varieties have been developed using sweet sorghum introductions, both germplasm bank accessions and landraces, many of them originally used for grain or forage production [28]. Genetic diversity studies can be very useful for sweet sorghum breeding programs, in which understanding the relationship between accessions is essential to define breeding strategies and to identify superior parents for the development of new cultivars [19,27,29–32].

Several strategies have been used to access genetic diversity in many crop species [33–37] based on morphological, agronomic, molecular, geographical and biochemical differences among accessions. Over the years, studies have dealt in estimating genetic diversity in cultivated sorghum based solely on phenotypic traits [38–40]. Even though phenotypic characterization provides a range of information about the genetic variability among accessions in a germplasm bank, the effects of environment, genotype-by-environment interaction, and measurement errors also contribute to the observed differences [41,42]. Thus, some authors have reported that the combined use of molecular markers and phenotypic traits could be advantageous to quantify the genetic differences among accessions [33,43,44]. However, few studies have assessed genetic diversity in sweet sorghum using morpho-agronomic traits and molecular markers simultaneously [32,45]. Wang et al. [32] accessed the genetic diversity of 142 sweet sorghum parent lines used in the hybrid breeding program of Heilongjiang Academy of Agricultural Sciences (Harbin, China) based on agronomical traits and simple sequence repeat (SSR) markers, and concluded that both tools should be considered simultaneously for the diversity analysis in hybrid breeding programs. Other studies have compared different types of cultivated sorghum using genetic diversity analyses based on molecular marker [19,29]. For example, Murray et al. [19] investigated the genetic relationship between sweet and grain sorghums using SSR and single nucleotide polymorphism (SNP) markers and Ritter et al. [29] used amplified fragment length polymorphism (AFLP) markers to access and to compare the level of genetic diversity between sweet and grain sorghums.

The use of molecular markers in genetic diversity analyses has some advantages over the phenotypic characterization, since molecular markers are not influenced by the environment and allow identification of differences in the DNA level that would be imperceptible via phenotyping [46–48]. Different molecular markers have been widely used to access genetic diversity in sorghum [27,35,49–53]. However, SNP markers have some advantages, for example local specificity, codominance, abundance along the genome, and potential for high throughput analysis. Recently, the costs and processing time were dramatically reduced by a variety of high-throughput SNP genotyping platforms, which offered the possibility of using abundant SNP markers as routine activities of breeding programs [42,54]. For example, genotyping-by-sequencing (GBS) [55] has provided new opportunities for breeders with cost-effective genome-wide scanning and multiplexed sequencing platforms [54,56]. Therefore, molecular markers are an excellent tool to efficiently assess the genetic diversity in a breeding program.

The aims of this study were: i) to perform phenotypic and molecular characterization of 100 sweet sorghum lines from the germplasm bank of the Embrapa Maize and Sorghum breeding program, using morpho-agronomic traits and SNP markers obtained via GBS; ii) to examine the relationship between the phenotypic and molecular diversity matrices; iii) and to infer about the population structure in the sorghum accessions.

## Material and methods

### Plant material

One hundred sweet sorghum accessions (S1 Table) from the germplasm bank of the Embrapa Maize and Sorghum breeding program were used. These sorghum accessions were classified as historical lines, modern lines and landraces according to the genealogy and historic background available in the GRIN (Germplasm Resources Information Network) database [57]. The historical lines (HL) are those developed and used between 1850 and the early 1900′s, and frequently have unknown origin and lack of concrete information about genealogy [19]. Modern lines (ML) correspond to those that have been genetically improved and have pedigree information. Landraces are those accessions collected in Africa and Asia that are often phenotypically diverse, but may exhibit some genetic similarity. The landraces were classified as LIS (Landrace World Collection—ICRISAT sorghum collection), LMN (Landrace Meridian Mississippi—USDA sorghum collection) and LSSM (Landrace Sorghum Seed Montpelier—CIRAD sorghum collection). The lines were not classified according to the races due to the inconsistent relationship previously detected between sweet sorghum and the traditional classification of major sorghum races.

### Phenotypic data

For phenotypic characterization, morphological traits related to the plant architecture, stem, leaf, panicle and caryopsis, and agro-industrial traits related to the production of sugars and biomass were used. The morphological traits were selected according to the list of *Sorghum bicolor* descriptors for cultivar registration purposes, based on the "Instructions for the Execution of Distinctness Tests, Homogeneity and Stability of Sorghum Cultivars" [58], which resulted in a total of 44 descriptors (S2 Table). Morphological characterization was performed in a greenhouse without replication, conducted at Embrapa Maize and Sorghum, in Sete Lagoas, State of Minas Gerais, Brazil (19° 28' 57'' south latitude and 44° 14' 48'' west longitude). Agro-industrial traits of economic importance for bioenergy production were characterized in a field experiment with one-hundred lines in a 10 x 10 lattice design with three replications, with plots of four rows of five meters (m) long and 0.70 m between rows. The following traits were evaluated: days to flowering (FLOW, in days after sowing) in which 50% of the plants in a plot started the pollen liberation; plant height (PH, in meters) as an average in each plot, measured from the soil surface to the top of the panicle; fresh biomass yield (FBY, in t.ha^-1^), weighing all plants from the effective plot area; juice extraction (EXT, in %), using hydraulic press, from five to eight plants sampled randomly per plot, without panicles; total soluble solids (TSS, in °Brix) in the extracted juice, using hydraulic press, with the use of a digital automatic refractometer; sucrose concentration in the juice (POL, in %), which is the measure of the amount of sucrose in the sugar mixture; reducing sugars in the juice (RSJ, in %), in which the weight of juice was calculated through the equation adapted from CONSECANA [59]; lignin (LIG, in %), hemicellulose (HEM, in %) and cellulose (CEL, in %) were measured following the sequential extraction method proposed by Van Soest and Wine [60], using samples of the stalk after juice extraction, which were dried in an oven for 72 hours at 65°C. The field experiment was planted in December 2013, in the experimental area of Embrapa Maize and Sorghum in Sete Lagoas, State of Minas Gerais, Brazil. The cultural treatments were those recommended for sweet sorghum crop [61].

### Molecular markers data

Leaf samples were collected from five plants per accession and the DNA extraction was performed using the Dneasy^®^ Plant Mini Kit (QIAGEN, Germantown, Maryland, USA). The quality and quantity of extracted DNA was checked in agarose gel and NanoDrop^®^ ND-1000 Spectrophotometer. Genotyping-by-sequencing (GBS) [55] was performed by the Institute for Genomic Diversity at Cornell University. Genomic DNA was digested individually with ApeKI, and the bar-coded DNA samples were pooled and sequenced in a HiSeq2000 platform (Illumina Inc., San Diego, California, USA). Sequencing data were separated for each accession and aligned to the BTx623 *Sorghum bicolor* reference genome [62, 63] version 2.1, using the Burrows-Wheeler Aligner (BWA) software [64]. SNPs were called using the GBS pipeline available in the software TASSEL [65]. Subsequently, SNP markers were filtered considering a minor allele frequency (MAF) of 5% and a maximum of 5% of missing genotypes per locus.

### Phenotypic analyses

For the morphological traits, a correlation analysis was performed based on the Pearson′s correlation coefficient [66] to identify highly correlated variables. Pairs of variables exhibiting correlation coefficient greater than 0.80 had one of the variables removed from the diversity analysis.

Agro-industrial phenotypic data were analyzed using the following mixed model, in which the number of days to flowering (FLOW) and the plant height (PH) were used as covariables:

$$y_{ijk}=\mu +\beta d_{ik}+\gamma h_{ik}+r_{k}+b_{jk}+g_{i}+\varepsilon_{ijk}$$

where *y*_*ijk*_ is the random phenotypic effect of the genotype *i* at block *j*, in replication *k*; *μ* is the general mean; *d*_*ik*_ is the number of days to flowering for the genotype *i*, in replication *k*, and *β* is the corresponding fixed effect; *h*_*ik*_ is the plant height for the genotype *i*, in replication *k*, and *γ* is the corresponding fixed effect; *r*_*k*_ is the fixed effect of replication *k*; *b*_*jk*_ is the random effect of block *j*, in replication *k*, with *b*_*jk*_~ $\text{N}(0,\sigma_{b}^{2})$; *g*_*i*_ is the random effect of genotype *i*, with *g*_*i*_ ~ $\text{N}(0,\sigma_{g}^{\text{2}})$; *ε*_*ijk*_ is a random non-genetic effect, with *ε*_*ijk*_ ~ N(0,*σ*^2^). FLOW and PH have direct effect on the phenotype of other agro-industrial traits related to the production of sugar and biomass. The correction for these phenological covariables was necessary for identify accessions that have favorable phenotypes for the production of sugar and biomass independently of their flowering time and plant height. Thus, this correction was considered when fitting the model for: fresh biomass yield (FBY), juice extraction (EXT), total soluble solids (TSS), sucrose concentration in the juice (POL), reducing sugars in the juice (RSJ), lignin (LIG), hemicellulose (HEM) and cellulose (CEL). Random and fixed effects in the model were tested using the likelihood ratio test (LRT) [67] and the Wald test [68], respectively, considering a 5% significance level. The adjusted means of each line for the agro-industrial traits were obtained via best linear unbiased predictor (BLUP) [69,70]. Variance components were estimated via residual maximum likelihood (REML) [71,72]. Heritabilities were calculated as:

$$h^{2}=\frac{\sigma_{G}^{2}}{\sigma_{G}^{2}+\frac{\sigma^{2}{}_{R}}{r}}$$

where $\sigma_{G}^{2}$ is the genetic variance; $\sigma^{2}{}_{R}$ is the residual variance; and *r* is the number of replications. All mixed models analyses were performed using the software GenStat v15 [73]. Then, phenotypic correlation between morphological and agro-industrial traits was estimated based on the Pearson's method, using the R package *Hmisc* (R Core Team 2015).

### Diversity analyses

Genetic diversity analyses in the sweet sorghum accessions were conducted separately using the phenotypic and the molecular data. Initially, for the phenotypic data, all morphological and agro-industrial variables were standardized. Then, the dissimilarity matrix between lines was calculated using the Average Euclidean distance [74]. The relative contribution of each morphological and agro-industrial trait for the diversity analysis was evaluated based on the Mahalanobis distance (D^2^), according to the method proposed by Singh [75], using the software Genes [76]. Subsequently, genetic distances between the sweet sorghum accessions were calculated based on the SNP data using the identity-by-state (IBS) coefficient [77] in the software TASSEL. This measure of similarity takes into account the number of identical alleles, whether or not inherited from a common ancestor. Based on the phenotypic and the molecular dissimilarity matrices, two separate cluster analyses were performed through the Neighbor-Joining method [78] using the software DARwin [79]. Different clusters of sweet sorghum accessions were identified according to the nodes present in the Neighbor-Joining trees. The Mantel test [80] was performed, using the software Genes, to test the significance of the correlation between the phenotypic and the molecular dissimilarity matrices, considering ten thousand random permutations and a 5% significance level. Averages of the agro-industrial traits were estimated for each cluster obtained through the phenotypic and the molecular diversity analysis, and were compared using the Duncan′s test [81] at a 5% significance level. In addition, a principal component analysis (PCA) [82] was performed, based on the molecular similarity matrix, in order to infer the population structure in the sweet sorghum accessions, using the package pcaMethods for the R software [83], available at the Bioconductor software [84].

## Results

### Phenotypic traits

**Fig 1:**
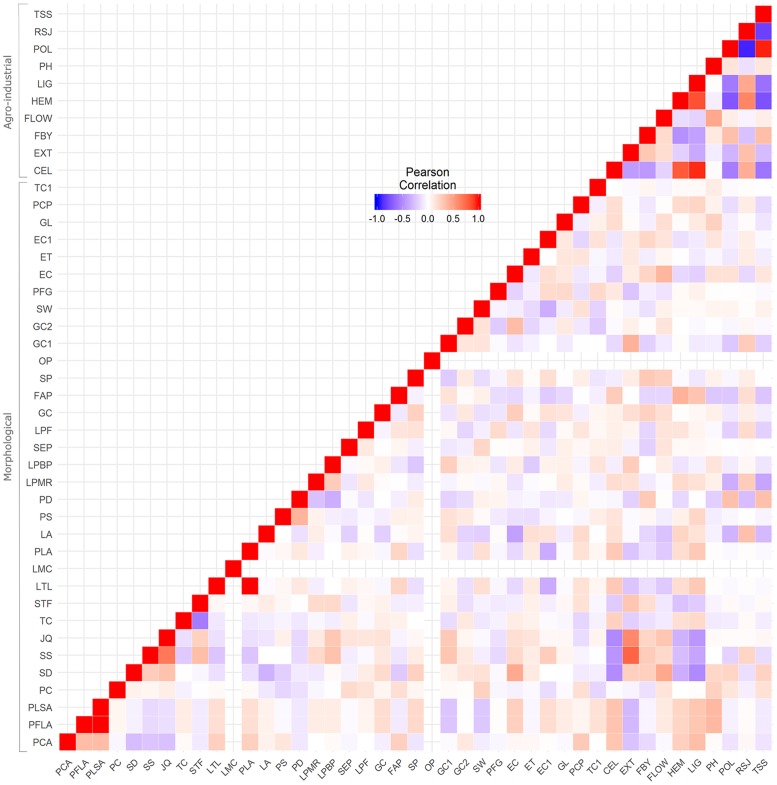
Heat map of phenotypic correlations among morphological and agro-industrial traits. The color assigned to a point in the heat map grid indicates the strength of a particular correlation between two traits. The level of correlation is indicated by red for positive correlations and blue for negative correlations, as depicted in the color key. PCA: pigmentation of the coleoptile by anthocyanin; PFLA: pigmentation of the first leaf by anthocyanin; PLSA: pigmentation of the leaf sheath by anthocyanin; PC: plant color; SD: stalk diameter; SS: stalk succulence; JQ: juice quality; TC: tillering capacity; STF: synchronization of tillering and flowering; LTL: length of the third leaf; PLA: pigmentation of the leaf by anthocyanin; LMC: leaf midrib color; LA: leaf angle; PS: panicle shape; PD: panicle density; LPMR: length of the panicle main rachis; LPBP: length of the primary branch of the panicle; SEP: shape and extension of the peduncle; LPF: length of the pedicelated flower; GC: glume color; FAP: formation of the awn in the palea; SP: stigma pigmentation; OP: ovary pigmentation; GC1: grain covering; GC2: grain color; SW: 1000-seed weight; PFG: presence of the forehead on the grain; EC: endosperm composition; ET: endosperm texture; EC1: endosperm color; GL: grain lustre; PCP: purple color on the pericarp; TC1: threshing capacity; CEL: cellulose; EXT: juice extraction; FBY: fresh biomass yield; FLOW: days to flowering; HEM: hemicellulose; LIG: lignin; PH: plant height; POL: sucrose concentration in juice; RSJ: reducing sugars in the juice and TSS: total soluble solids.

After correlation analysis performed for the 44 morphological traits, 11 variables that showed high correlation with another variable, were not included in the diversity analysis (r > 0.80). The remaining 33 descriptors used in the diversity analysis are listed in the S2 Table, where additional information about all morphological traits is presented. Most of the correlations between the 33 descriptor traits were very low and not significantly different from zero (Fig 1). Only a few pairs of descriptors exhibited correlations greater than 0.3 and significantly different from zero, considering a 5% significance level, for example: PCA/PFLA (0.35); PCA/PLSA (0.35); SD/JQ (0.32); SS/JQ (0.68); SS/LPBP (0.32); SS/STF (0.34) and SD/EC (0.44) (S3 Table).

Genetic variances were significant, using the likelihood ratio test at a 1% significance level, for all agro-industrial traits (Table 1), indicating the existence of genetic variability among the 100 sweet sorghum lines. The variance of blocks was not significant for the fresh biomass yield (FBY) and the reducing sugars in the juice (RSJ). The fixed effects in the model were tested using the Wald test. The phenological covariable days to flowering (FLOW) was significant for the following response variables: fresh biomass yield (FBY), total soluble solids (TSS, °Brix), sucrose concentration in the juice (POL), hemicellulose (HEM) and RSJ (Table 1). Plant height (PH) was only significant as a phenological covariable for FBY and cellulose (CEL) and the fixed effect of replication was only significant for the total soluble solids (TSS) and the sucrose concentration in the juice (POL). The heritability varied from 0.62 to 0.92 for RSJ and FLOW, respectively (Table 1). According to the results of the correlation between morphological and agro-industrial traits (Fig 1) the highest values of correlation, considering a 5% significance level (S3 Table), were observed among the agro-industrial traits, for example: CEL/HEM (0.8); CEL/LIG (0.95); POL/TSS (0.97); HEM/RSJ (0.62); POL/RSJ (-0.93) and RSJ/TSS (-0.82).

**Table 1:** Fixed and random effects, heritability, average, minimum and maximum phenotypic values for the agro-industrial traits.

	Fixed effects			Random effects						
	r_k_	*d*_*ik*_	h_ik_	$\sigma_{g}^{2}$	$\sigma_{b}^{2}$	$\sigma_{e}^{2}$	Average	Minimum	Maximum	*h*^2^
CEL	NS	NS	*	6.61**	0.49**	3.75	36.85	29.76	45.85	0.84
EXT	NS	NS	NS	31.48**	4.80**	13.37	61.68	35.98	79.52	0.88
FBY	NS	**	**	65.30**	NS	114.50	46.62	11.71	89.14	0.63
FLOW	NS	-	-	42.86**	2.01**	11.46	77.70	56.00	95.00	0.92
HEM	NS	*	NS	1.17**	1.01**	1.86	21.86	17.70	27.17	0.65
LIG	NS	NS	NS	0.48**	0.12**	0.36	5.81	3.65	8.85	0.80
PH	NS	-	-	0.11**	0.02**	0.06	2.74	1.50	3.80	0.85
POL	*	*	NS	2.59**	0.40**	2.48	8.76	1.51	13.47	0.76
RSJ	NS	*	NS	0.03**	NS	0.06	1.50	0.57	2.98	0.62
TSS	**	*	NS	2.52**	0.59**	2.49	13.76	6.70	14.10	0.75

*r*_*k*_ is the fixed effect of replication *k*; *d*_*ik*_ is the fixed effect of the covariable number of days to flowering for the genotype *i* in replication *k*; *h*_*ik*_ is the fixed effect of the covariable plant height for the genotype *i* in replication *k*; $\sigma_{g}^{2}$ is the genetic variance; $\sigma_{b}^{2}$ is the variance of the effect of blocks within replications; $\sigma_{e}^{2}$ is the non-genetic variance; CEL: cellulose (%); EXT: juice extraction (%); FBY: fresh biomass yield (t.ha^-1^); FLOW: days to flowering (in days after sowing); HEM: hemicellulose (%); LIG: lignin (%); PH: plant height (m); POL: sucrose concentration in juice (%); RSJ: reducing sugars in the juice (%); TSS: total soluble solids (°Brix); *h*^2^: heritability;

**, * and NS: significant at 5%, 1% of significance level and non-significant, respectively, in the Wald test and the likelihood ratio test (LRT) for fixed and random effects, respectively.

### Molecular markers

After raw GBS sequence data processing, a total of 403,433 SNP markers distributed along the ten sorghum chromosomes were obtained, varying from 21,823 to 71,557 SNPs for the chromosomes 8 and 1, respectively (S4 Table). Then, SNP data was filtered for a minor allele frequency (MAF) of 5% and a maximum of 5% of missing genotypes per locus, resulting in a total of 40,206 polymorphic SNPs, which varied from 2,327 to 7,019 SNPs for the chromosomes 8 and 1, respectively. The final chromosome coverage varied from 55.34 to 77.62 Mbp for the chromosomes 8 and 2, respectively, with an average of 65.81 Mbp per chromosome. The average marker density was one SNP for every 18 kb, with the highest chromosome saturation observed for the chromosome 1 (1 SNP/10,503 bp), and the lowest SNP marker density observed for the chromosome 5 (1 SNP/25,657 bp) (S4 Table).

### Genetic diversity

**Fig 2:**
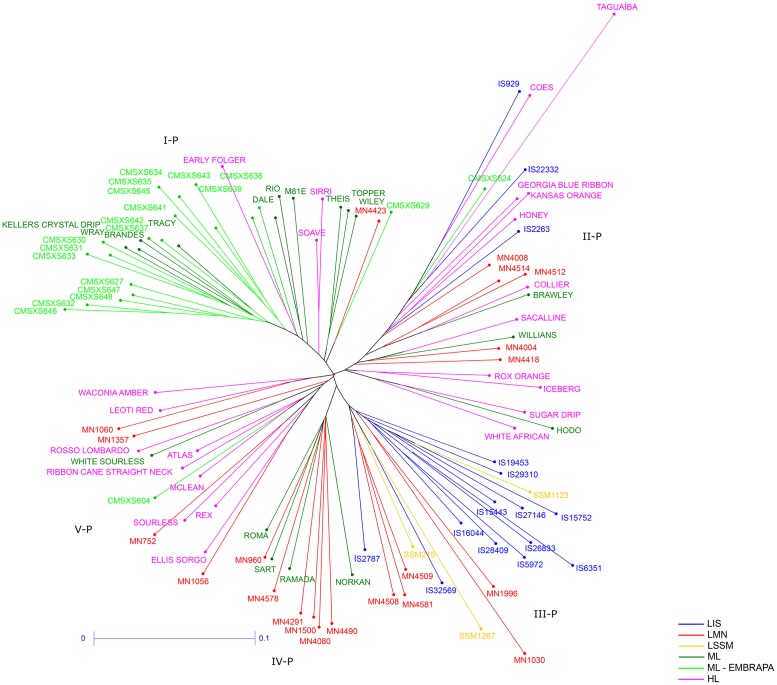
Neighbor-Joining tree using phenotypic data. Euclidean distances between the sweet sorghum accessions were calculated based on the standardized phenotypic data. The colors of the branches correspond to the six subpopulations defined according to the genealogy and the historic background of the sweet sorghum lines. I-P, II-P, III-P, IV-P and V-P correspond to the clusters identified through the Neighbor-Joining method. LIS: Landrace World Collection—ICRISAT sorghum collection; LMN: Landrace Meridian Mississippi—USDA sorghum collection; LSSM: Landrace Sorghum Seed Montpelier—CIRAD sorghum collection; ML: Modern Line; ML—EMBRAPA: Modern Line EMBRAPA; and HL: Historical Line. The scale-bar (0–0.1) represents the coefficient of dissimilarity.

Based on the morphological and the agro-industrial traits, the Neighbor-Joining method resulted in the identification of five major clusters of sweet sorghum lines (Fig 2). The cluster I-P was the most homogeneous off all clusters and consisted of 32 lines, mostly CMSXS lines derived from the Embrapa Maize and Sorghum breeding program, except for CMSXS624 and CMSXS604, which have different parents than the other CMSXS lines and grouped in the clusters II-P and V-P, respectively. The lines Theis, Wray, Brandes and Rio, which were used as parents of most of these CMSXS lines (see S1 Table), were also grouped in the cluster I-P. CMSXS627 and Keller Crystal Drip exhibited a high relationship, which is in agreement with the CMSXS627 genealogy. Dale, considered a modern line, grouped together with one of its parents, Tracy. Most of the lines grouped in the cluster I-P were classified as modern lines, except for the landrace MN4423 and the historical lines Early Folger, Soave and Sirri. The cluster II-P was the most heterogeneous of all clusters, in which most of the lines do not have information about genealogy and historic background, and consisted of 23 lines: 11 historical lines, 3 modern lines, 1 modern line Embrapa (CMSXS624), 3 landraces IS and 5 landraces MN. In this cluster, Taguaíba is a historical line collected in Brazil probably introduced from Africa, which grouped relatively distant from the other lines due to the fact that it did not flower in consequence of the photoperiod during cultivation. Most of the landraces IS and all the landraces SSM were grouped in the cluster III-P. This cluster consisted of 20 lines: 12 landraces IS, 3 landraces SSM and 5 landraces MN. The cluster IV-P was the smallest one, with only 10 lines: 4 modern lines (Sart, Ramada, Roma and Norkan) and 6 landraces MN. The cluster V-P was also heterogeneous with 15 lines: 9 historical lines, 1 modern line (White Sourless), 1 modern line Embrapa (CMSXS604) and 4 landraces MN. The morphological traits that greatly contributed to the diversity study were: leaf angle (8.61%), juice quality (8.21%), plant color (7.66%), pigmentation coleoptile by anthocyanin (6.29%), stalk succulence (6.11%), stalk diameter (5.43%), grain color (3.74%), glume color (3.54%) and panicle shape (3.51%), with a total contribution of 53.1%. The agro-industrial traits that greatly contributed were: juice extraction (12.07%), cellulose (11.39%), plant height (11.33%), reducing sugars (10.47%), sucrose concentration in the juice (10.38%), total soluble solids (10.35%), lignin (9.38%), days to flowering (9.17%), fresh biomass yield (7.9%) and hemicellulose (7.59%).

**Fig 3:**
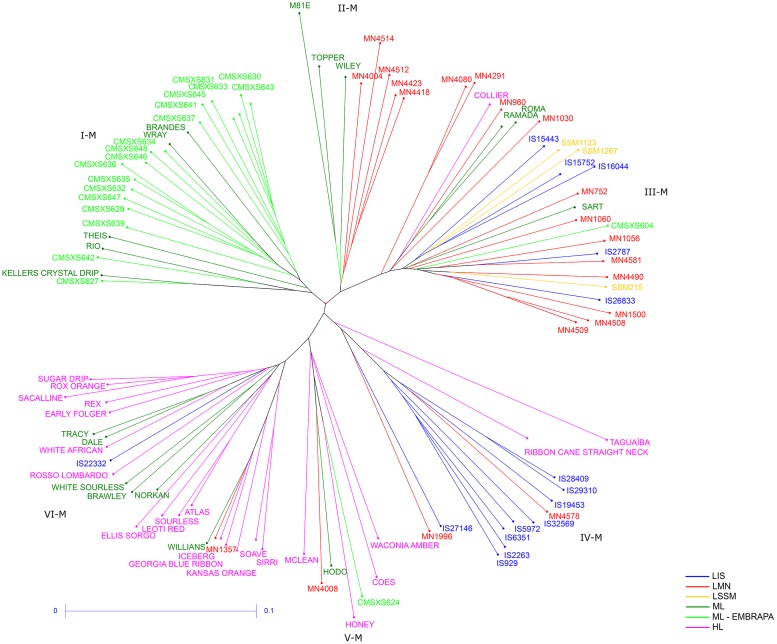
Neighbor-Joining tree using SNP data. Genetic distances between the sweet sorghum accessions were calculated using the identity-by-state (IBS) coefficient. The colors of the branches correspond to the six subpopulations defined according to the genealogy and the historic background of the sweet sorghum lines. I-M, II-M, III-M, IV-M, V-M and VI-M correspond to the clusters identified through the Neighbor-Joining method. LIS: Landrace World Collection—ICRISAT sorghum collection; LMN: Landrace Meridian Mississippi—USDA sorghum collection; LSSM: Landrace Sorghum Seed Montpelier—CIRAD sorghum collection; ML: Modern Line; ML—EMBRAPA: Modern Line EMBRAPA; and HL: Historical Line. The scale-bar (0–0.1) represents the coefficient of dissimilarity.

The Neighbor-Joining method, using the SNP markers data, resulted in 6 major clusters of sweet sorghum lines (Fig 3). The cluster I-M consisted of 23 lines with a composition very similar to the cluster I-P, including most of the CMSXS lines, except for CMSXS604 and CMSXS624 that grouped in the clusters III-M and V-M, respectively. Wray, Brandes, Rio and Theis were also grouped in this cluster. CMSXS627 and Keller Crystal Drip exhibited a high genetic relationship as also observed in the cluster I-P. The cluster II-M consisted of a small number of lines: 3 modern lines and 5 landraces MN. The cluster III-M was the most heterogeneous and consisted of 25 lines, including 5 landraces IS (IS15443, IS15752, IS16044, IS2787 and IS26833), some landraces MN, all landraces SSM, 1 historical line (Collier), 3 modern lines (Roma, Ramada e Sart) and a modern line Embrapa (CMSXS604). Most of the landraces IS grouped in the cluster IV-M, which consisted of 13 lines, including 2 landraces MN and 2 historical lines. The cluster V-M consisted of 7 lines: 4 historical lines, 1 modern line (Hodo), 1 modern line Embrapa (CMSXS624) and 1 landrace MN (MN4008). Most of the historical lines were grouped in the cluster VI-M, which consisted of 24 lines. Dale and Norkan, considered as modern lines, also grouped in this cluster with one of its parents Tracy and Atlas, respectively. Other modern lines (Williams, White Sourless and Brawley) and 1 landrace IS (IS2232) were also in the group VI-M.

The phenotypic and the molecular diversity matrices exhibited a low correlation coefficient (0.35, significant at a 1% significance level) obtained via the Mantel Test, which is in agreement with the inconsistencies observed between the clusters formed by the phenotypic and the molecular diversity analyses. The clusters obtained by the molecular diversity analysis were more consistent with the genealogy and the historic background of the sweet sorghum accessions than the clusters obtained through the phenotypic diversity analysis.

**Fig 4:**
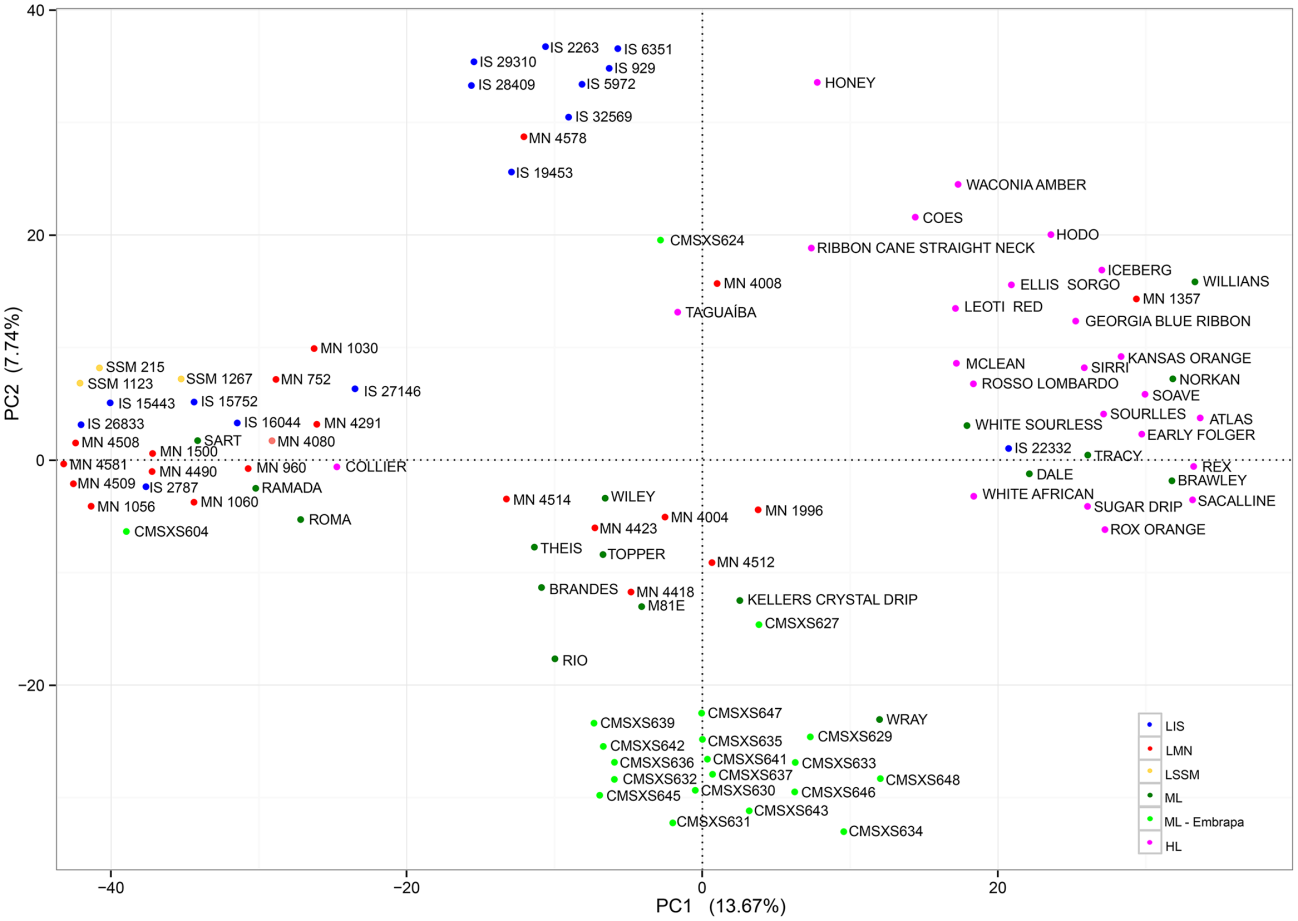
Principal component analysis using SNP data. Plotting the first two principal components (PC1 and PC2) using SNP data. The colors of the genotypes correspond to the six subpopulations of sweet sorghum according to the genealogy and the historic background. LIS: Landrace World Collection—ICRISAT sorghum collection; LMN: Landrace Meridian Mississippi—USDA sorghum collection; LSSM: Landrace Sorghum Seed Montpelier—CIRAD sorghum collection; ML: Modern Line; ML—EMBRAPA: Modern Line EMBRAPA; and HL: Historical Line.

The population structure revealed by the principal component analysis (PCA) based on the SNP markers data was also consistent with the genealogy and the historic background of the sweet sorghum lines (Fig 4). In this analysis, the first (PC1) and second (PC2) principal components explained 13.67% and 7.74%, respectively, of the genetic variability observed in the sweet sorghum lines (Fig 4). As expected, the PCA results were more consistent with the clusters obtained by the Neighbor-Joining method using the SNP markers data when compared to the phenotypic data.

**Fig 5:**
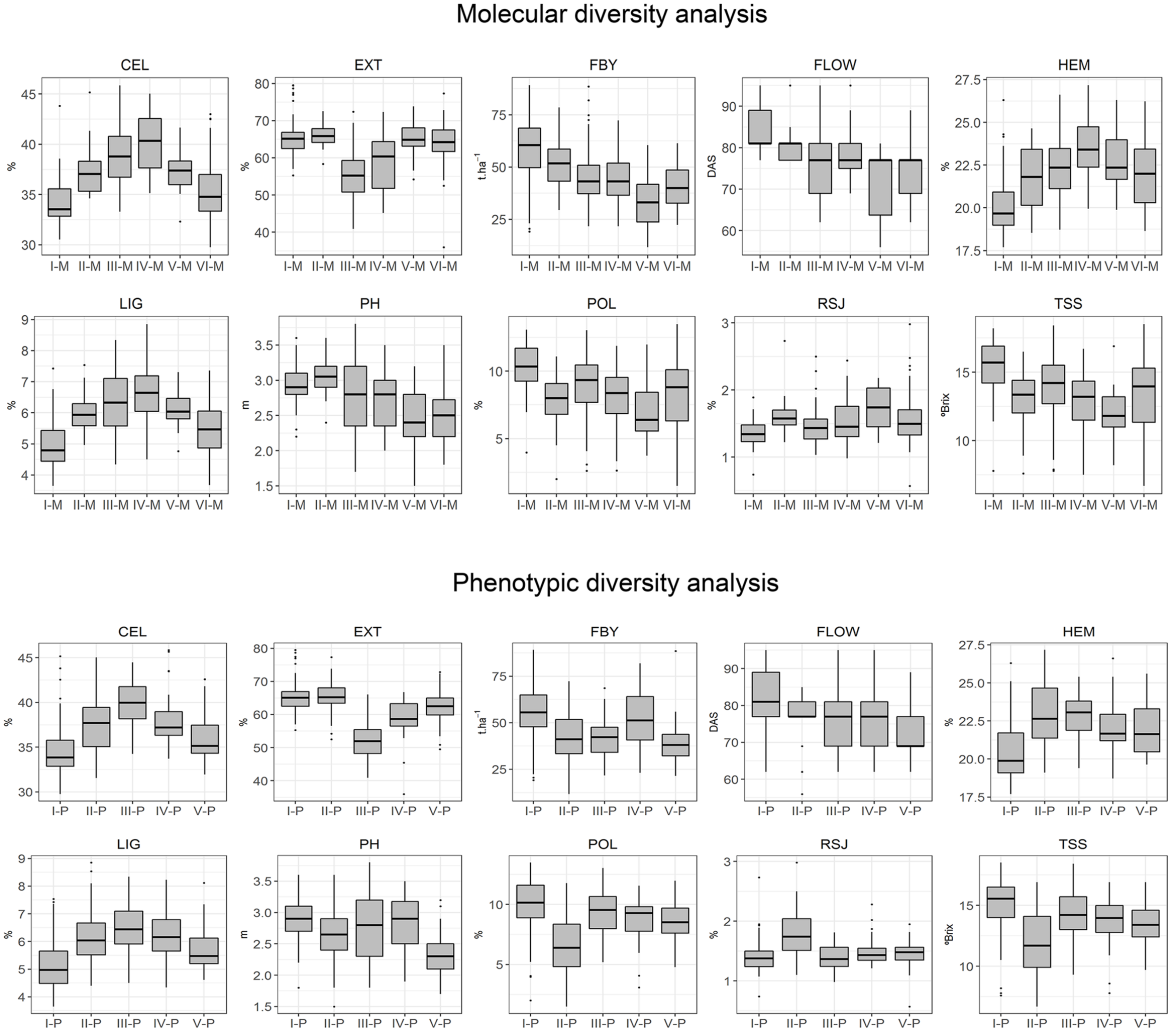
Boxplot analysis showing the distribution of agro-industrial traits according to each cluster identified through molecular and phenotypic diversity analysis. The upper, median, and lower quartiles of gray boxes represent the 75^th^, 50^th^, and 25^th^ percentiles of the clusters, respectively. The vertical lines represent the variation of the clusters. Dots represent outliers. CEL: cellulose; EXT: juice extraction; FBY: fresh biomass yield; FLOW: days to flowering; HEM: hemicellulose; LIG: lignin; PH: plant height; POL: sucrose concentration in juice; RSJ: reducing sugars in the juice and TSS: total soluble solids.

The distribution of the agro-industrial traits were showed for all clusters obtained through the molecular and the phenotypic diversity analysis, respectively (Fig 5). For most of the traits, significant differences were observed between averages of distinct clusters (S5 Table). According to the averages of the agro-industrial traits obtained for each cluster formed by the molecular diversity analysis (S5 Table), the groups III-M and VI-M exhibited significantly different averages and interesting phenotypes for several agro-industrial traits. For example, averages of 49.12 and 46.26 t.ha^-1^ of fresh biomass yield, 60.37 and 63.06% of juice extraction, 13.99 and 14.30 °Brix of total soluble solids, 9.08 and 8.30% of sucrose concentration, 1.44 and 1.56% of reducing sugars in the juice were observed for the clusters II-M and VI-M, respectively. On the other hand, based on the phenotypic diversity analysis, the clusters III-P and V-P showed satisfactory consistency with the lines genealogy and the historic background and also interesting averages for several agro-industrial traits. For example, averages of 44.74 and 51.36 t.ha^-1^ of fresh biomass yield, 57.57 and 65.49% of juice extraction, 14.31 and 16.01 °Brix of total soluble solids, 9.45 and 9.03% of sucrose concentration, 1.69 and 1.46% of reducing sugars in the juice were observed for the clusters III-P and V-P, respectively (S5 Table). Besides the interesting phenotypes for bioenergy production, these clusters formed by the molecular (II-M and IV-M) and the phenotypic (III-P and V-P) diversity analyses exhibited considerable genetic divergences with the I-M and I-P clusters, respectively, which were composed by most of the CMSXS (Embrapa) sweet sorghum lines. Thus, these results of the molecular and the phenotypic diversity analyses can be combined and used to identify potential lines to be introduced in the Embrapa Maize and Sorghum breeding program focusing on bioenergy.

## Discussion

Genetic diversity and population structure analyses in this collection of sweet sorghum accessions provided important information to define breeding strategies and to identify superior parents for the development of new sorghum cultivars focusing on bioenergy production. The clusters obtained by the molecular diversity analysis were more consistent with the genealogy and the historic background of the sweet sorghum accessions than the clusters formed by the phenotypic diversity analysis. SNP markers have revealed valuable information about the relationship among the sweet sorghum accessions, especially for those with unknown genealogy and historic background, allowing the identification of potential parents to be used in the Embrapa Maize and Sorghum breeding program focusing on bioenergy production. The lack of consistency between the clusters identified by the phenotypic diversity analysis and the genealogy and the historic background of the sweet sorghum accessions can be attributed to the large genotype-by-environment interaction effect commonly observed for morphological and agro-industrial traits of quantitative inheritance. Therefore, molecular markers combined with the phenotypic characterization of sweet sorghum accessions should be used to investigate the genetic diversity of potential lines to be introduced in a breeding program.

The low correlation between the phenotypic and the molecular diversity matrices should not be considered as a limitation to access the genetic diversity but as an indicative of the complementarity of these tools [46,48,85]. Most of the variation detected by molecular markers is commonly of the non-adaptive type and therefore not subject to natural and/or artificial selection, different from the phenotypic traits which are mostly subject to natural and/or artificial selection [43]. Several studies have also reported a lack of consistency between phenotypic and molecular distances in different species, such as pepper [48], cotton [85], wheat [49,86], maize [87,88], barley [89], ryegrass [90] and *Avena sterilis* [91]. In sorghum, some authors have found low correlations between the genetic distances estimated by molecular markers and by phenotypic traits [32,51]. For example, Geleta et al. [51] conducted a genetic diversity study in a collection of 45 sorghum accessions, using morphological data and SSR markers, and found a low but significant correlation (*r* = 0.19, *p* < 0.01) between the phenotypic and the molecular diversity matrices. These authors stated that it is possible to obtain a relevant minimum subset of markers that can be used in combination with morphological data to better classify genotypes. Furthermore, their study indicated that, although the phenotypic characterization is time-consuming and greatly influenced by the environment, in general, it is a significant and practical way to make progress in the evaluation of sorghum germplasm. Wang et al. [32] conducted a genetic diversity study using 142 sweet sorghum lines and also found a low but significant correlation (r = 0.45, *p* < 0.01) between molecular and phenotypic diversity matrices, concluding that the clusters of accessions formed based on the SSR markers data did not coincide with the clusters based on the phenotypic data, suggesting that the molecular diversity analysis provided better results. According to Geleta et al. [51] and Singh et al. [43], the best way to identify divergence among genotypes is the combined use of phenotypic and molecular data, since these tools provide complementary results.

Breeding populations exhibiting high genetic variability are required for the success in selecting individuals with favorable genotypes for a given trait [30]. The knowledge about the genetic relationship among inbred lines is useful to maintain the genetic variability as well as to identify promising parental combinations to create segregating populations in a breeding program [92]. According to the averages of agro-industrial traits, lines grouped in different clusters identified through the molecular diversity analysis, with favorable phenotypes for bioenergy production and exhibiting considerable genetic divergences with the CMSXS lines, could be suggested as potential parents to be introduced in the development of improved lines and/or hybrids in the Embrapa Maize and Sorghum breeding program. For example, the following sweet sorghum inbred lines may be interesting to these purposes: Georgia Blue Ribbon, Rosso Lombardo, Atlas, Ellis Sorgo, Rex, Sourless, MN4509, MN4508, MN1030, MN4581, SSM1123, IS15443, IS15752, IS2787 and IS16044.

Expressive contribution of the morphological traits was observed (53.1%) to the phenotypic diversity analysis, and these traits represent a simple way of measuring genetic diversity while studying genotype performance under normal growing conditions [41]. Moreover, most of the morphological traits used in this study have a qualitative inheritance, whose expression is not strongly influenced by the environment [93]. Other studies also highlighted the contribution of morphological traits to diversity analyses in sweet sorghum. For example, Gerrano et al. [93] used six AFLP primer combinations and nine qualitative morphological traits to study the genetic diversity in 17 sorghum accessions, and concluded that the morphological traits were able to distinguish accessions and that the molecular markers complemented the analysis to separate closely related individuals. Grenier et al. [94] evaluated 45 sorghum accessions using ten qualitative morphological traits, and observed a wide morphological diversity in the evaluated germplasm, which contributed to cluster the genotypes according to each geographic region of Ethiopia and Eritrea.

Some sweet sorghum accessions used in this study were also previously used in other genetic diversity studies [19, 30]. For example, 33 out of the 125 sweet sorghum accessions evaluated by Murray et al. [19] were also used in this study, and presented similar clustering patterns. The accessions Brandes, Keller, M81E, Rio, Wiley, Wray and Sart (Modern Lines) were grouped in the same cluster as also observed through the molecular diversity analysis performed in this study. Moreover, the accessions MN1056, MN960, MN1060, MN1500 (Landraces MN), Iceberg, Ellis Sorgo, Mclean, Kansas Orange, Atlas, Sugar Drip, White African and Sacalline (Historical Lines) were also grouped in the same cluster by Murray et al. [19]. Similar clustering patterns were also observed by Ali et al. [30] for the accessions Dale, Tracy, White African, Kansas Orange, Rox Orange, Williams, Iceberg, Early Folger, Rio, Keller, Roma and Ramada.

Accessing the genetic diversity of potential parental lines by phenotypic and molecular characterization can provide valuable information in order to help breeders to identify promising crosses in a commercial hybrid breeding program [95]. Sweet sorghum has emerged as an ideal feedstock for bioethanol production to exploit alternative bioenergy. Indeed, significant genetic potential exists in the sweet sorghum germplasm collection [31]. The wide genetic variability observed in this study for brix, sucrose concentration in the juice, stalk and biomass yield indicate a high potential for the development of high-yielding sweet-stalked high-sucrose sweet sorghum lines [9]. Breeders can select parental lines grouped in different phenotypic and molecular clusters to perform higher heterotic crosses, since it is expected to occur higher levels of heterosis between clusters than within clusters [32]. This study also indicated that the assessment of the genetic diversity using molecular markers is indispensable and complementary to the phenotypic characterization. Thus, in order to obtain improved sweet sorghum hybrids with high level of heterosis, breeders should simultaneously select parental lines from different clusters based on agro-industrial traits and molecular marker data.

## Conclusion

Phenotypic and molecular characterization revealed the existence of considerable genetic variability between the sweet sorghum accessions from the Embrapa Maize and Sorghum breeding program. The clusters obtained by the molecular diversity analysis were more consistent with the genealogy and the historic background of the sweet sorghum accessions than the clusters identified in the phenotypic diversity analysis. The population structure revealed by the PCA based on the SNP markers data was consistent with the genealogy, the historic background of the sweet sorghum lines, and, as expected, with the clusters obtained by the Neighbor-Joining method using the SNP markers data. A low correlation was observed between the molecular and the phenotypic diversity matrices, which highlight the complementarity between the molecular and the phenotypic characterization to assist a breeding program.

## Supporting information

S1 Table Sweet sorghum lines used for phenotypic and molecular characterization. Additional information is provided for each accession according to the source, pedigree, place of origin and registration year. Accessions were classified as LIS (Landrace World Collection—ICRISAT sorghum collection), LMN (Landrace Meridian Mississippi—USDA sorghum collection), LSSM (Landrace Sorghum Seed Montpelier—CIRAD sorghum collection), ML (Modern Line), ML—EMBRAPA (Modern Line EMBRAPA) and HL (Historical Line).
(DOCX)

S2 Table Morphological descriptors used for phenotypic characterization. Morphological descriptors were selected according to the list of *Sorghum bicolor* descriptors for cultivar registration purposes, based on the “Instructions for the Execution of Distinctness Tests, Homogeneity and Stability of Sorghum Cultivars”.
(DOCX)

S3 Table Pearson's correlation coefficient among morphological and agro-industrial traits. (XLSX)

S4 Table SNP markers used for molecular characterization. Number of SNPs before and after data filtering for a minor allele frequency (MAF) of 5% and a maximum of 5% of missing genotypes per locus, final chromosome coverage and final marker density.
(DOCX)

S5 Table Averages and standard deviations (SD) of the agro-industrial traits obtained for each cluster identified through molecular and phenotypic diversity analyses. (DOCX)

## References

[1] Jonker JGG, van der Hilst F, Junginger HM, Cavalett O, Chagas MF, Faaij APC. Outlook for ethanol production costs in Brazil up to 2030, for different biomass crops and industrial technologies. Appl Energy. 2015; 173: 494–510. doi: 10.1016/j.apenergy.2015.01.090

[2] Calviño M, Messing J. Sweet sorghum as a model system for bioenergy crops. Curr Opin in Biotech. 2012; 23:323–329. doi: 10.1016/j.copbio.2011.12.002 22204822 10.1016/j.copbio.2011.12.002

[3] Khatiwada D, Leduc S, Silveira S, McCallum I. Optimizing ethanol and bioelectricity production in sugarcane biorefineries in Brazil. Renew Energy. 2016; 85: 371–386. doi: 10.1016/j.renene.2015.06.009

[4] Scott V, Stuart Haszeldine R, Tett B SF, Oschlies A. Fossil fuels in a trillion tonne world. Nat Publ Gr. 2015; 5: 419–423. doi: 10.1038/nclimate2578

[5] Chen X, Nuñez HM, Xu B. Explaining the reductions in Brazilian sugarcane ethanol production costs: Importance of technological change. GCB Bioenergy. 2015; 7:468–478. doi: 10.1111/gcbb.12163

[6] Mullet J, Morishige D, McCormick R, Truong S, Hilley J, McKinley B, Anderson R, Rooney W. Energy Sorghum-A genetic model for the design of C4 grass bioenergy crops. J of Exp Bot. 2014; 13:3479–3489. doi: 10.1093/jxb/eru229 24958898 10.1093/jxb/eru229

[7] Regassa TH, Wortmann CS. Sweet sorghum as a bioenergy crop: Literature review. Biom and Bioen. 2014; 64:38–355. doi: 10.1016/j.biombioe.2014.03.052

[8] Olukoya IA, Bellmer D, Whiteley JR, Aichele CP. Evaluation of the environmental impacts of ethanol production from sweet sorghum. Energy Sustain Dev. 2015; 24:1–8. doi: 10.1016/j.esd.2014.10.004

[9] Reddy BV, Ramesh S, Reddy PS, Ramaiah B, Salimath P, Kachapur R. Sweet Sorghum—A Potential Alternate Raw Material for Bio-ethanol and Bio-energy. Int Sorghum Millets Newsl. 2005; 46:79–86.

[10] Fernandes G, Braga TG, Fischer J, Parrella RAC, de Resende MM, Cardoso VL. Evaluation of potential ethanol production and nutrients for four varieties of sweet sorghum during maturation. Renew Energy. 2014; 71:218–524. doi: 10.1016/j.renene.2014.05.033

[11] Ying G, Hu S, Li Y, Chen D, Zhu B, Smith KM. Optimization and analysis of a bioethanol agro-industrial system from sweet sorghum. Renew Energy. 2010; 35:2902–2909 doi: 10.1016/j.renene.2010.04.024

[12] Burks PS, Felderhoff TJ, Viator HP, Rooney WL. The Influence of Hybrid Maturity and Planting Date on Sweet Sorghum Productivity during a Harvest Season. Agron J. 2013;105: 263–267. doi: 10.2134/agronj2012.0317

[13] Winberry JJ. The Sorghum Syrup Industry: 1854–1975. Agric Hist Soc. 1980;54: 343–352.

[14] Doggett H. Sorghum. Longmans, Green and Co. Ltd, London; 1970.

[15] Sherwood SF. Starch in Sorghum Juice. Ind Eng Chem. 1923;15: 727–728.

[16] Smith CW, Frederiksen RA. Sorghum: origin, history, technology and production. Wiley; 2000.

[17] Freeman KC and Broadhead DM. Culture of sweet sorghum for syrup production USDA agricultural handbook; 1973 441p.

[18] Hunter EL A I. Sweet sorghum. Hortic Reviews. 1997; 21:73–104.

[19] Murray SC, Rooney WL, Hamblin MT, Mitchell SE, Kresovich S. Sweet Sorghum Genetic Diversity and Association Mapping for Brix and Height. The Plant Gen. 2009; 2:48–62. doi: 10.3835/plantgenome2008.10.0011

[20] Fernandes FT and RE Schaffert. Sorghum in Brazil, Sorghum Diseases. 1978; 15–17.

[21] Parrella RAC. Genetic breeding of sorghum. Agroe in J. 2001; 2 (3): 8–9.

[22] Brazil, Ministry of Agriculture, Livestock and Food Supply S of P and A-E. National Agroenergy Plan 2006–2011. 2006.

[23] Schaffert RE, Santos FG, Borgonovi RA, Silva JB. Learn to plant sweet sorghum. Agroq. 1980; 13:10–14.

[24] Mace ES, Tai S, Gilding EK, Li Y, Prentis PY, Bian L, Campbell BC, Hu W, Innes DJ, Han X, Cruickshank A. Whole-genome sequencing reveals untapped genetic potential in Africa’s indigenous cereal crop sorghum. Nat Commun. 2013;4 doi: 10.1038/ncomms3320 23982223 10.1038/ncomms3320 PMC3759062

[25] Morris GP, Ramu P, Deshpande SP, Hash CT, Shah T, Upadhyaya HD, et al Population genomic and genome-wide association studies of agroclimatic traits in sorghum. Proceedings of the National Academy of Sciences. 2013; 110 (2) 453–458. 10.1073/pnas.1215985110 PMC3545811 23267105

[26] Harlan JR and deWet JMJ. A simplified classification of cultivated sorghum. Crop Sci. 1972; 12: 172–176. doi: 10.2135/cropsci1972.0011183X001200020005x

[27] Elangovan M, Kiran babu P, Seetharama N, Patil J V. Genetic Diversity and Heritability Characters Associated in Sweet Sorghum [*Sorghum bicolor* (L.) Moench]. Sugar Tech. 2014; 16:200–210. doi: 10.1007/s12355-013-0262-5

[28] Corn RJ. Heterosis and composition of sweet sorghum. Texas A&M University. 2009.

[29] Ritter KB, McIntyre CL, Godwin ID, Jordan DR, Chapman SC. An assessment of the genetic relationship between sweet and grain sorghums, within Sorghum bicolor ssp. bicolor (L.) Moench, using AFLP markers. Euph. 2007; 157: 161–167. doi: 10.1007/s10681-007-9408-4

[30] Ali ML, Rajewski JF, Baenziger PS, Gill KS, Eskridge KM, Dweikat I. Assessment of genetic diversity and relationship among a collection of US sweet sorghum germplasm by SSR markers. Mol Breed. 2008; 21:497–509. doi: 10.1007/s11032-007-9149-z

[31] Wang ML, Zhu C, Barkley NA, Chen Z, Erpelding JE, Murray SC, et al Genetic diversity and population structure analysis of accessions in the US historic sweet sorghum collection. Theor Appl Genet. 2009; 120: 13–23. doi: 10.1007/s00122-009-1155-6 19760215 10.1007/s00122-009-1155-6

[32] Wang L, Jiao S, Jiang Y, Yan H, Su D, Sun G, et al Genetic diversity in parent lines of sweet sorghum based on agronomical traits and SSR markers. Field Crop Res. 2013; 149:11–19. doi: 10.1016/j.fcr.2013.04.013

[33] Franco J, Crossa J, Ribaut JM, Bertran J, Warburton ML, Khairallah M. A method for combining molecular markers and phenotypic attributes for classifying plant genotypes. Theor Appl Genet. 2001; 103:944–952. doi: 10.1007/s001220100641

[34] Mohammadi SA, Prasanna BM. Analysis of Genetic Diversity in Crop Plants-Salient Statistical Tools and Considerations Sampling Strategies. Crop Sci. 2003; 43:1235–1248. doi: 10.2135/cropsci2003.1235

[35] Smith JSC, Kresovich S, Hopkins MS, Mitchell SE, Dean RE, Woodman WL, Lee M P K. Genetic diversity among elite sorghum inbred lines assessed with simple sequence repeats. Crop Sci. 2000; 40: 226–232. doi: 10.2135/cropsci2000.401226x

[36] Ghebru B, Schmidt RJ, Bennetzen JL. Genetic diversity of Eritrean sorghum landraces assessed with simple sequence repeat (SSR) markers. Theor Appl Genet. 2002; 105: 229–236. doi: 10.1007/s00122-002-0929-x 12582524 10.1007/s00122-002-0929-x

[37] Chiorato AF, Carbonell SAM, Benchimol LL, Chiavegato MB, Dias LAS, Colombo CA. Genetic diversity in common bean accessions evaluated by means of morpho-agronomical and RAPD data. Sci Agr. 2007; 64:256–262. doi: 10.1590/S0103-90162007000300007

[38] Zongo JD, Gouyon PH, Sandmeier M. Genetic variability among sorghum accessions from the Sahelian agroecological region of Burkina Faso. Biodivers Conserv. 1993; 2:627–636. doi: 10.1007/BF00051963

[39] Appa RS, Prasada Rao KE, Mengesha MH R V. Morphological diversity in sorghum germplasm from India. Genet Resour Crop Evol. 1996; 43: 559–567. doi: 10.1007/BF00138832

[40] Ayana A, Bekele E. Geographical patterns of morphological variation in sorghum (*Sorghum bicolor* (L.) *Moench*) germplasm from Ethiopia and Eritrea: qualitative characters. Hered. 1998;129: 195–205.

[41] Van Beuningen LT and Busch RH. Genetic diversity among North American spring wheat cultivars: III Cluster analysis based on quantitative morphological traits. Crop Sci. 1997;37: 981–988. doi: 10.2135/cropsci1997.0011183X003700030046x

[42] Schlötterer C. The evolution of molecular markers—just a matter of fashion? Nat Rev Genet. 2004;5: 63–69. doi: 10.1038/nrg1249 14666112 10.1038/nrg1249

[43] Singh SP, Nodari R, Gepts P, Singh SP. (1991) Genetic Diversity in Cultivated Common Bean: I. Allozymes. Crop Sci. 1991; 31:19–23. doi: 10.2135/cropsci1991.0011183X003100010004x

[44] Barro-Kondombo C, Sagnard F, Chantereau J, Deu M, vom Brocke K, Durand P, et al Genetic structure among sorghum landraces as revealed by morphological variation and microsatellite markers in three agroclimatic regions of Burkina Faso. Theor Appl Genet. 2010; 120:1511–1523. doi: 10.1007/s00122-010-1272-2 20180097 10.1007/s00122-010-1272-2

[45] Lekgari A, Dweikat I. Assessment of Genetic Variability of 142 Sweet Sorghum Germplasm of Diverse Origin with Molecular and Morphological Markers. Open J Ecol. 2014;4: 371–393. doi: 10.4236/oje.2014.47034

[46] Marić S, Bolarić S, Martinčić J, Pejić I, Kozumplik V. Genetic diversity of hexaploid wheat cultivars estimated by RAPD markers, morphological traits and coefficients of parentage. Plant Breed. 2004; 123:366–369. doi: 10.1111/j.1439-0523.2004.00956.x

[47] Lammerts van Bueren ET, Backes G, de Vriend H, Østergård H. The role of molecular markers and marker assisted selection in breeding for organic agriculture. Euph. 2010; 175:51–64. doi: 10.1007/s10681-010-0169-0

[48] Lefebvre V, Goffinet B, Chauvet JC, Caromel B, Signoret P, Brand R, et al Evaluation of genetic distances between pepper inbred lines for cultivar protection purposes: Comparison of AFLP, RAPD and phenotypic data. Theor Appl Genet. 2001; 102:741–750. doi: 10.1007/s001220051705

[49] Yang W, Oliveira AC, Godwin I, Schertz K, Bennetzen JL. Comparison of DNA marker technologies in characterizing plant genome diversity: variability in Chinese sorghums. Crop Sci. 1996;36: 1669–1676. doi: 10.2135/cropsci1996.0011183X003600060042x

[50] Djè Y, Heuertz M, Lefe´bvre C V X. Assessment of genetic diversity within and among germplasm accessions in cultivated sorghum using microsatellite markers. Theor Appl Genet. 2000;100: 918–925. doi: 10.1007/s001220051371

[51] Geleta N, Labuschagne MT, Viljoen CD. Genetic diversity analysis in sorghum germplasm as estimated by AFLP, SSR and morpho-agronomical markers. Biodivers Conserv. 2006; 15: 3251–3265. doi: 10.1007/s10531-005-0313-7

[52] Billot C, Ramu P, Bouchet S, Chantereau J, Deu M, Gardes L, et al Massive Sorghum Collection Genotyped with SSR Markers to Enhance Use of Global Genetic Resources. PLoS One. 2013; 8:e59714 doi: 10.1371/journal.pone.0059714 23565161 10.1371/journal.pone.0059714 PMC3614975

[53] Lekgari A, Dweikat I. Assessment of Genetic Variability of 142 Sweet Sorghum Germplasm of Diverse Origin with Molecular and Morphological Markers. Open J Ecol. 2014;4: 371–393. doi: 10.4236/oje.2014.47034

[54] Varshney RK, Nayak SN, May GD, Jackson SA. Next-generation sequencing technologies and their implications for crop genetics and breeding. Trends in Biot. 2009 27: 522–530. doi: 10.1016/j.tibtech.2009.05.006 19679362 10.1016/j.tibtech.2009.05.006

[55] Elshire RJ, Glaubitz JC, Sun Q, Poland JA, Kawamoto K, Buckler ES, et al A robust, simple genotyping-by-sequencing (GBS) approach for high diversity species. PLoS One. 2011; 6:e19379 doi: 10.1371/journal.pone.0019379 21573248 10.1371/journal.pone.0019379 PMC3087801

[56] Kim C, Guo H, Kong W, Chandnani R, Shuang LS, Paterson AH. Application of genotyping by sequencing technology to a variety of crop breeding programs. Plant Sci. 2016 242: 14–22. doi: 10.1016/j.plantsci.2015.04.016 26566821 10.1016/j.plantsci.2015.04.016

[57] GRIN—Germplasm Resources Information Network [Internet]. http://www.ars-grin.gov

[58] Brazil, Ministry of Agriculture L and FS. Instructions for implementation of distinctness tests, homogeneity and stability of sorghum (Sorghum bicolor). 1997.

[59] Consecana—Conselho dos Produtores de Cana de açúcar, Açúcar e Etanol do Estado de São Paulo. Manual de instruções. 2006.

[60] Van Soest PJ, RH Wine. Determination of lignin and cellulose in acid-detergent fiber with permanganate. 1968; 51: 780–785.

[61] Borgonovi RA, Giacomini SF, Santos HL dos, Ferreira A da S, Waquil JM, Silva JB da, Cruz I. Recommendations for sweet sorghum planting. Sete Lagoas: Embrapa Milho e Sorgo, 1982. 16 p.

[62] Paterson AH, Bowers JE, Bruggmann R, Dubchak I, Grimwood J, Gundlach H, Haberer G, Hellsten U, Mitros T, Poliakov A, Schmutz J, Spannagl M, Tang H, Wang X, Wicker T, Bharti AK, Chapman J, Feltus FA, Gowik U, Grigoriev IV, Lyons E, Maher CA, Martis M, Narechania A, Otillar RP, Penning BW, Salamov AA, Wang Y, Zhang L, Carpita NC, Freeling M, Gingle AR, Hash CT, Keller B, Klein P, Kresovich S, McCann MC, Ming R, Peterson DG, Mehboob-ur-Rahman, Ware D, Westhoff P, Mayer KF, Messing J, Rokhsar DS. The Sorghum bicolor genome and the diversification of grasses Nat. 2009; 29:551–6. doi: 10.1038/nature07723 19189423 10.1038/nature07723

[63] Goodstein DM, Shu S, Howson R, Neupane R, Hayes RD, Fazo J, et al Phytozome: A comparative platform for green plant genomics. Nucleic Acids Res. 2012; 40: 1178–1186. doi: 10.1093/nar/gkr944 22110026 10.1093/nar/gkr944 PMC3245001

[64] Li H, Durbin R. Fast and accurate long-read alignment with Burrows-Wheeler transform. Bioinf. 2010; 26: 589–595. doi: 10.1093/bioinformatics/btp698 20080505 10.1093/bioinformatics/btp698 PMC2828108

[65] Bradbury PJ, Zhang Z, Kroon DE, Casstevens TM, Ramdoss Y, Buckler ES. TASSEL: Software for association mapping of complex traits in diverse samples. Bioinf. 2007; 23: 2633–2635. doi: 10.1093/bioinformatics/btm308 17586829 10.1093/bioinformatics/btm308

[66] Pearson K. Regression, hereditary and panmixia. Philos Trans R Soc London Ser A. 1896; 187: 253–318.

[67] Neyman J, Pearson ES. On the Use and Interpretation of Certain Test Criteria for Purposes of Statistical Inference: Part I Source Biometrika Biometrika Trust. Oxford University Press; 1928; 20: 175–240.

[68] Wald A. Tests of statistical hyphotheses concerning sereval parameters when the number of observations is large. T Am Math Soc. 1943; 3:426–481.

[69] Henderson CR. Best Linear Unbiased Estimation and Prediction under a Selection Model. Biom. 1975; 31: 423–447. 1174616

[70] Bernardo R. Breeding for quantitative traits in plants Hardbound: Stemma; 2010 369 p.

[71] Patterson HD T R. Recovery of inter-block information when block sizes are unequal. Biom. 1971; 58: 545–554.

[72] Harville DA. Maximum Likelihood Approaches to Variance Component Estimation and to Related Problems. Source J Am Stat Assoc. American Statistical Association; 1977; 72: 320–338.

[73] Payne R, Murray D, Harding S, Baird D, Soutar D. Introduction to GenStat for Windows ^®^ TM (15 Edition) th.http://www.genstat.co.uk/

[74] Bussab WO, Miazak ES AD. Introdução à análise de agrupamento. In: Simpósio Nacional de Probabilidade e Estatística. São Paulo: Simpósio Nacional de Probabilidade e Estatística; 1990. 105p.

[75] Singh D. The relative importance of characters affecting genetic divergence. Indian Journals. 1981; 41: 237–245.

[76] Cruz CD. Programa Genes—Aplicativo computacional em genética e estatística. Viçosa: Universidade Federal de Viçosa; 2008 http://arquivo.ufv.br/dbg/genes/gdown1.htm

[77] Powell JE, Visscher PM, Goddard ME. Reconciling the analysis of IBD and IBS in complex trait studies. Nat Publ Gr. 2010; 11:800–805. doi: 10.1038/nrg2865 20877324 10.1038/nrg2865

[78] Saitou N and Nei M. The Neighbor-Joining method: a new method for reconstructing phylogenetic trees. Mol Biol Evol. 1987; 4: 406–425. 3447015 10.1093/oxfordjournals.molbev.a040454

[79] Perrier X and Jacquemoud-Collet JP. DARwin software [Internet]; 2006. http://darwin.cirad.fr/darwin

[80] Mantel N. The Detection of Disease Clustering and a Generalized Regression Approach. Cancer Res. 1967; 27: 209–220. 6018555

[81] Duncan DB. Multiple range and multiple F tests. Biom. 1955; 11: 1–42.

[82] Price AL, Patterson NJ, Plenge RM, Weinblatt ME, Shadick NA, Reich D. Principal components analysis corrects for stratification in genome-wide association studies. Nat Genet. 2006; 38:904–909. doi: 10.1038/ng1847 16862161 10.1038/ng1847

[83] R Core Team. The R project for statistical computing-R version 3.1.1 Software. [Internet]. 2015 Available: http://www.r-project.org

[84] Stacklies W, Redestig H, Scholz M, Walther D, Selbig J. pcaMethods—A bioconductor package providing PCA methods for incomplete data. Bioinf. 2007; 23:1164–1167. doi: 10.1093/bioinformatics/btm069 17344241 10.1093/bioinformatics/btm069

[85] Rana MK, Singh VP, Bhat K V. Assessment of genetic diversity in upland cotton (Gossypium hirsutum L.) breeding lines by using amplified fragment length polymorphism (AFLP) markers and morphological characteristics. Genet Resour Crop Evol. 2005; 52:989–997. doi: 10.1007/s10722-003-6113-6

[86] Soriano JM, Villegas D, Aranzana MJ, Garcia Del Moral LF, Royo C. Genetic structure of modern durum wheat cultivars and mediterranean landraces matches with their agronomic performance. PLoS One. 2016; 11:8:e0160983 doi: 10.1371/journal.pone.0160983 27513751 10.1371/journal.pone.0160983 PMC4981446

[87] Hartings H, Berardo N, Mazzinelli GF, Valoti P, Verderio A, Motto M. Assessment of genetic diversity and relationships among maize (*Zea mays* L.) Italian landraces by morphological traits and AFLP profiling. Theor Appl Genet. 2008; 117:831–42. doi: 10.1007/s00122-008-0823-2 18584146 10.1007/s00122-008-0823-2

[88] Rebourg C, Gouesnard B, Welcker C, Dubreuil P, Chastanet M C A. Maize introduction into Europe: the history reviewed in the light of molecular data. Theor Appl Genet. 2003;106:895–903. doi: 10.1007/s00122-002-1140-9 12647065 10.1007/s00122-002-1140-9

[89] Schut JW, Qi X, Stam P. Association between relationship measures based on AFLP markers, pedigree data and morphological traits in barley. Theor Appl Genet. 1997; 95:1161–1168.

[90] Roldán-Ruiz I, Dendauw J, Van Bockstaele E, Depicker A, De Loose M. AFLP markers reveal high polymorphic rates in ryegrasses (*Lolium spp*.). Mol Breed. 2000; 6:125–134. doi: 10.1023/A:1009680614564

[91] Beer SC, Gofreda J P T. Assessment of genetic variation in *Avena sterilis* using morphological traits, isozymes and RFLPs. Crop Sci. 1993; 33:1386–1393. doi: 10.2135/cropsci1993.0011183X003300060051x

[92] Becelaere GV, Lubbers EL, Paterson AH, Chee PW. Pedigree-vs. DNA marker-based genetic similarity estimates in Cotton. Crop Sci. 2005; 45:2281–2287. doi: 10.2135/cropsci2004.0715

[93] Gerrano AS, Labuschagne MT, Van Biljon A, Shargie NG. Genetic diversity assessment in sorghum accessions using qualitative morphological and amplified fragment length polymorphism markers. Sci. agric. 2014; 71:394–401. doi: 10.1590/0103-9016-2013-0251

[94] Grenier C, Bramel PJ, Dahlberg JA, El-Ahmadi A, Mahmoud M, Peterson GC, Rosenow DT, Ejeta G. Sorghums of the Sudan: analysis of regional diversity and distribution. Genet Resour Crop Evol. 2004; 51: 489–500. doi: 10.1023/B:GRES.0000024155.43149.71

[95] Segovia-Lerma A, Murray LW, Townsend MS, Ray IM. Population-based diallel analyses among nine historically recognized alfalfa germplasms. Theor Appl Genet. 2004; 109:1568–1575. doi: 10.1007/s00122-004-1784-8 15372154 10.1007/s00122-004-1784-8

