# Supplementary material for: Phenotypic and molecular characterization of sweet sorghum accessions for bioenergy production
Source: PLoS One. 2017 Aug 17;12(8):e0183504. doi: 10.1371/journal.pone.0183504 (PMC5560702; doi:10.1371/journal.pone.0183504)
Supplement: S1 Table — Additional information is provided for each accession according to the source, pedigree, place of origin and registration year. Accessions were classified as LIS (Landrace World Collection—ICRISAT sorghum collection), LMN (Landrace Meridian Mississippi—USDA sorghum collection), LSSM (Landrace Sorghum Seed Montpelier—CIRAD sorghum collection), ML (Modern Line), ML—EMBRAPA (Modern Line EMBRAPA) and HL (Historical Line). (DOCX) [file pone.0183504.s001.docx]

**S1 Table. Sweet sorghum lines used for phenotypic and molecular characterization.** Additional information is provided for each accession according to the source, pedigree, place of origin and registration year. Accessions were classified as LIS (Landrace World Collection - ICRISAT sorghum collection), LMN (Landrace Meridian Mississippi - USDA sorghum collection), LSSM (Landrace Sorghum Seed Montpelier - CIRAD sorghum collection), ML (Modern Line), ML - EMBRAPA (Modern Line EMBRAPA) and HL (Historical Line).

| **Lines** | **Source** | **Classification** | **Pedigree/Background information** | **Place of origin/Registration year** | **Reference** |
| --- | --- | --- | --- | --- | --- |
| ATLAS | MISSISSIPPI | HL | Blackhull Kafir x Sourless | 1938 | [1] Schertz et al. (1990) |
| BRANDES | MISSISSIPPI | ML | Collier 706-C x MN 1500 | 1968 | [2] Coleman and Broadhead,1968 |
| BRAWLEY | USDA-ARS | ML | Rex x White-seeded Collier | 1983 | [3] USDA, 1958 |
| CMSXS604 | EMBRAPA | ML | NP3R x PU785091 | Brazil,1975 | - |
| CMSXS624 | EMBRAPA | ML | CMSXS605 x CMSXS607 | Brazil, 1975 | - |
| CMSXS627 | EMBRAPA | ML | Derived from F2 progeny of cross MER 50-1 x Rio | Brazil, 1979 | - |
| CMSXS629 | EMBRAPA | ML | Theis x Wray | Brazil, 1986 | - |
| CMSXS630 | EMBRAPA | ML | Theis x Wray | Brazil, 1983 | - |
| CMSXS631 | EMBRAPA | ML | Brandes x Wray | Brazil, 1983 | - |
| CMSXS632 | EMBRAPA | ML | Brandes x Wray | Brazil, 1983 | - |
| CMSXS633 | EMBRAPA | ML | Brandes x Wray | Brazil, 1983 | - |
| CMSXS634 | EMBRAPA | ML | Theis x Wray | Brazil, 1983 | - |
| CMSXS635 | EMBRAPA | ML | Theis x Wray | Brazil, 1983 | - |
| CMSXS636 | EMBRAPA | ML | Theis x Wray | Brazil, 1983 | - |
| CMSXS637 | EMBRAPA | ML | Theis x Wray | Brazil, 1983 | - |
| CMSXS639 | EMBRAPA | ML | Theis x Wray | Brazil, 1983 | - |
| CMSXS641 | EMBRAPA | ML | Brandes x Wray | Brazil, 1984 | - |
| CMSXS642 | EMBRAPA | ML | Rio x Wray | Brazil, 1984 | - |
| CMSXS643 | EMBRAPA | ML | Brandes x Wray | Brazil, 1984 | - |
| CMSXS645 | EMBRAPA | ML | Brandes x Wray | Brazil, 1984 | - |
| CMSXS646 | EMBRAPA | ML | Theis x Wray | Brazil, 1984 | - |
| CMSXS647 | EMBRAPA | ML | Theis x Wray | Brazil, 1984 | - |
| CMSXS648 | EMBRAPA | ML | Theis x Wray | Brazil, 1984 | - |
| COES | USDA-ARS | HL | - | - | - |
| COLLIER | MISSISSIPPI | HL | - | Introduced from South Africa, 1946 | - |
| DALE | MISSISSIPPI | ML | Tracy x MN960 | Mississipi, 1970 | [4] Broadhead et al., 1970 |
| EARLY FOLGER | MISSISSIPPI | HL | - | Introduced from Africa, 1942 | - |
| ELLIS SORGO | USDA-ARS | HL | Leoti x Atlas | 1948 | [5] Karper, 1949 |
| GEORGIA BLUE RIBBON  **TABELA 1.** Materiais de sorgo sacarino, pertencentes ao Programa de Melhoramento Genético da Embrapa Milho e Sorgo, selecionados para a caracterização fenotípica e molecular (continua) | MISSISSIPPI | HL | - | - | [6] Freeman et al., 1973 |
| HODO | USDA-ARS | ML | - | - | - |
| HONEY | USDA-ARS | HL | - | Accession collected in India, 1983 | [7] Freeman et al., 1986 |
| ICEBERG | USDA-ARS | HL | - | 1983 | - |
| IS15443 | ICRISAT | LIS | - | Cameroon, Central Africa, 1973 | - |
| IS15752 | ICRISAT | LIS | - | Cameroon, Central Africa, 1972 | - |
| IS16044 | ICRISAT | LIS | - | Cameroon, Central Africa, 1972 | - |
| IS19453 | ICRISAT | LIS | - | Botswana, Southern Africa | - |
| IS22332 | ICRISAT | LIS | - | Botswana, Southern Africa,1980 | - |
| IS2263 | ICRISAT | LIS | - | Sudan, East Africa, 1993 | - |
| IS26833 | ICRISAT | LIS | - | Sudan, East Africa, 1993 | - |
| IS27146 | ICRISAT | LIS | - | Zimbabwe, Southern Africa | - |
| IS2787 | ICRISAT | LIS | - | Kenya, East Africa, 1967 | - |
| IS28409 | ICRISAT | LIS | - | Yemen, Asia | - |
| IS29310 | ICRISAT | LIS | - | Swaziland, Southern Africa | - |
| IS32569 | ICRISAT | LIS | - | Somalia, East Africa | - |
| IS5972 | ICRISAT | LIS | - | India, Asia, 1967 | - |
| IS6351 | ICRISAT | LIS | - | India, Asia, 1967 | - |
| IS929 | ICRISAT | LIS | - | Sudan, East Africa, 1993 | - |
| KANSAS ORANGE | USDA-ARS | HL | - | Introduced from Africa, 1850´s | - |
| KELLERS CRYSTAL DRIP | USDA-ARS | ML | Mer. 50-1 x Rio | Texas, USA, 1961 | [8] Broadhead et al., 1979 |
| LEOTI RED | USDA-ARS | HL | - | Texas, USA, 1946 | [9] Swanson and Laude, 1934 |
| M81E | MISSISSIPPI | ML | Brawley x (Brawley x Rio) | 1983 | [10] Broadhead et al., 1981 |
| MCLEAN | USDA-ARS | HL | - | - | - |
| MN1357 | USDA-ARS | LMN | - | - | - |
| MN 1030 | USDA-ARS | LMN | - | Sudan, East Africa, 1945 | - |
| MN1056 | USDA-ARS | LMN | Selection of SC 1055 | Sudan, East Africa | [11] Freeman, 1979 |
| MN1060 | USDA-ARS | LMN | - | Sudan, East Africa | [11] Freeman, 1979 |
| MN1500 | USDA-ARS | LMN | Selection of SC 1057 | Uganda,East Africa | [12] Kresovich et al., 1988 |
| MN1996 | USDA-ARS | LMN | - | Malawi, East Africa, 1946 | - |
| MN4004 | USDA-ARS | LMN | - | North of Australia | - |
| MN4008  **TABELA 1.** Materiais de sorgo sacarino, pertencentes ao Programa de Melhoramento Genético da Embrapa Milho e Sorgo, selecionados para a caracterização fenotípica e molecular (continua) | USDA-ARS | LMN | - | 1980 | - |
| MN4080 | USDA-ARS | LMN | - | Zaire, Southern Africa, 1958 | - |
| MN4291 | USDA-ARS | LMN | - | 1979 | - |
| MN4418 | USDA-ARS | LMN | - | Soviet Union, 1965 | - |
| MN4423 | USDA-ARS | LMN | - | Australia, 1964 | - |
| MN4490 | USDA-ARS | LMN | - | Ethiopia, East Africa | - |
| MN4508 | USDA-ARS | LMN | - | Uganda, East Africa, 1960 | - |
| MN4509 | USDA-ARS | LMN | - | Uganda, East Africa, 1960 | - |
| MN4512 | USDA-ARS | LMN | - | India, Asia | - |
| MN4514 | USDA-ARS | LMN | - | India, Asia | - |
| MN4578 | USDA-ARS | LMN | - | Ethiopia, East Africa | - |
| MN4581 | USDA-ARS | LMN | - | Ethiopia, East Africa | - |
| MN752 | USDA-ARS | LMN | - | Sudan, East Africa | - |
| MN960 | USDA-ARS | LMN | - | Sudan, East Africa | [11] Freeman, 1979 |
| NORKAN | USDA-ARS | ML | Atlas x Early Sumac | 1941 | - |
| RAMADA | USDA-ARS | ML | Mer, 45-45 x (MN 1056 x MN 1054 x MN 1060) | - | [13] Freeman et al., 1974 |
| REX | USDA-ARS | HL | - | - | [14] Sherwood, 1923 |
| RIBBON CANE STRAIGHT NECK | USDA-ARS | HL | - | - | - |
| RIO | USDA-ARS | ML | REX x MN 1048 | 1965 | [15] Broadhead, 1972 |
| ROMA | USDA-ARS | ML | Mer 45-45 X MN 1060 | - | - |
| ROSSO LOMBARDO | - | HL | - | Buenos Aires, Argentina, 1959 | - |
| ROX ORANGE | MISSISSIPPI | HL | - | 1938 | - |
| SACCALLINE | USDA-ARS | HL | - | - | [16] Vinall et al., 1936 |
| SART | USDA-ARS | ML | - | Sudan, East Africa, 1951 | [17] Stokes et al., 1951 |
| SIRRI | USDA-ARS | HL | - | Sudan, East Africa | - |
| SOAVE | USDA-ARS | HL | - | Sudan, East Africa | - |
| SOURLESS | MISSISSIPPI | HL | - | - | - |
| SSM1123 | CIRAD | LSSM | - | Níger, Western Africa | - |
| SSM1267 | CIRAD | LSSM | - | Cameroon, Central Africa | - |
| SSM215 | CIRAD | LSSM | - | Ethiopia, East Africa | - |
| SUGAR DRIP | USDA-ARS | HL | - | Belongs to group Orange | [7] Freeman et al., 1986 |
| TAGUAÍBA | - | HL | - | Amazon Region, Brazil, 1979 | - |
| THEIS | USDA-ARS | ML | Wiley/C,PSpecial x (MN 1054/White African/MN660) | 1978 | - |
| TOPPER | USDA-ARS | ML | Mer60-2 x Brandes | 1995 | - |
| TRACY | USDA-ARS | ML | White African (Mer. 51-2)/Sumac | 1953 | [18] Stokes et al., 1953 |
| WACONIA AMBER | USDA-ARS | HL | - | - | - |
| WHITE AFRICAN | MISSISSIPPI | HL | - | South of Africa, 1938 | - |
| WHITE SOURLESS | USDA-ARS | ML | - | Collected in Texas, USA 1961 | - |
| WILEY | USDA-ARS | ML | (Collier/ MN 822) x MN 2046 | 1965 | [19] Coleman et al., 1956 |
| WILLIAMS | USDA-ARS | ML | - | - | [6] Freeman et al., 1973 |
| WRAY | MISSISSIPPI | ML | Mer, 57-1 x Brawley x Rio | 1981 | [20] Broadhead et al., 1978 |

EMBRAPA: Embrapa Maize and Sorghum, Sete Lagoas, Brazil; USDA-ARS: United States Department of Agriculture/Agricultural Research Service, United States; ICRISAT: International Crops Research Institute for the Semi-Arid Tropics, Índia; CIRAD*: Centre de Coopération Internationale en Recherche Agronomique pour le Développement*; MISSISSIPPI: Sugar Crops Field Station, Meridian, Mississippi, United States.

**References cited in S1 Table:**

1. Schertz K, Stec A DJ. Isozyme genotypes of sorghum lines and hybrids in the United States. Agr Expt Stn. 1990; 1719:15.
2. Coleman OH and DM Broadhead. Brandes - a new variety of sweet sorghum for sirup production. State College M, editor. 1968.
3. USDA. Government Printing Ofce. Brawley - a new high-sugar sorgo variety developed for irrigated areas. 1958.
4. Broadhead, D.M., O.H. Coleman and KCF. Dale - a new variety of sweet sorghum for sirup production. 1970.
5. Karper RE. Registration of sorghum varieties. Agron J. 1949; 41: 536-540.
6. Freeman KC and Broadhead DM. Culture of sweet sorghum for syrup production. USDA agricultural handbook; 1973. 441p.
7. Freeman KC DB and NZ. Sweet sorghum culture and sirup production. USDA Agriculture Handbook; 1986.
8. Broadhead DM KF and NZ. Keller - a new high-sucrose sweet sorghum with potential for sugar production. 1979.
9. Swanson A and Laude H. Varieties of sorghum in Kansas. 1934.
10. Broadhead DM KF and NZ. M81E - a new variety of sweet sorghum. 1981.
11. Freeman KC. Germplasm release of sweet sorghum lines with resistance to downy mildew, leaf anthracnose and rust and with adequate combining ability to produce progeny with agronomic characters acceptable for commercial sirup and/or sugar production. 1979; 4:2.
12. Kresovich S, Miller FR, Dominy RE, Monk RL, Broadhead DM. Registration of “Grassl” sweet sorghum. Crop Sci. 1988; 28: 194-195.
13. Freeman KC, WR Cowley, BA Smith, N Zummo, L Reyes, DM Broadhead DR and KS. Ramada - a new variety of sweet sorghum for potential sugar production in south Texas. 1974.
14. Sherwood SF. Starch in Sorghum Juice. Ind Eng Chem. 1923;15: 727-728.
15. Broadhead DM. Registration of “Rio” sweet sorghum. Crop Sci. 1972;12:716.
16. Vinall HN, Stephens JC, Agronomist A, Martin JH. Identification, history and distribution of common sorghum varieties. Senior Agronomist Division of Forage Crops and Diseases Senior Agronomist Division of Cereal Crops and Diseases Bureau of Plant Industry. 1936; Technical Bulletin, 506.
17. Stokes IE, OH Colman JO. Sart - a new variety of sorgo for sirup production in Mississippi. 1951.
18. Stokes IE, OH Colman JO. Tracy - a new mid-season variety of sorgo for sirup production in Mississippi. 1953.
19. Coleman OH, IE Stokes, KC Freeman, JL Dean, JF O’Kelly, SP Crockett, R Kuykendall, BC Hurt, TE Ashley, CL Blount, WL Giles PH and RA. Wiley - a new variety of sorgo for syrup production in Mississippi. 1956.
20. Broadhead DM KF and NZ. Wray - a new variety of sweet sorghum for sugar production. 1978.
