# Supplementary material for: Phenotypic and molecular characterization of sweet sorghum accessions for bioenergy production
Source: PLoS One. 2017 Aug 17;12(8):e0183504. doi: 10.1371/journal.pone.0183504 (PMC5560702; doi:10.1371/journal.pone.0183504)
Supplement: S2 Table — Morphological descriptors were selected according to the list of Sorghum bicolor descriptors for cultivar registration purposes, based on the “Instructions for the Execution of Distinctness Tests, Homogeneity and Stability of Sorghum Cultivars”. (DOCX) [file pone.0183504.s002.docx]

**S2 Table. Morphological descriptors used for phenotypic characterization**. Morphological descriptors were selected according to the list of *Sorghum bicolor* descriptors for cultivar registration purposes, based on the “Instructions for the Execution of Distinctness Tests, Homogeneity and Stability of Sorghum Cultivars”.

| **Morphological trait** | **Description** | **Phenotype** |
| --- | --- | --- |
| PCA* | Pigmentation of the coleoptile by anthocyanin | 1-Absent  2-Present |
| PFLA* | Pigmentation of the first leaf by anthocyanin | 1-Null  2-Intermediate  3-Intense |
| PLSA* | Pigmentation of the leaf sheath by anthocyanin | 1-Absent  2-Present |
| PC* | Plant color | 1-Straw  2-Red  3-Purple |
| SD* | Stalk diameter | 1-Small  2-Middle  3-Large |
| SS* | Stalk succulence | 1-Dry  2-Succulent |
| JQ* | Juice quality | 1-Insipid  2-Sweet |
| TC* | Tillering capacity | 1-Null  2-Low  3-High |
| STF* | Synchronization of tillering and flowering | 1-Coincident  2-Non-coincident |
| LIS | Leaf insertion on the stalk | 1-With ligule  2-Without ligule |
| LMC1 | Leaf margin curling | 1-Curl  2-Flat |
| LTL* | Length of the third leaf | 1-Short  2-Intermediate  3-Long |
| WTL | Width of the third leaf | 1-Narrow  2-Intermediate  3-Long |
| PLA* | Pigmentation of the leaf by anthocyanin | 1-Light Green  2-Medium green  3-Dark Green |
| LMC* | Leaf midrib colour | 1-White  2-Green  3-Yellow  4-Brown |
| SW | Sheath waxy | 1-Absent  2-Present |
| LA* | Leaf angle | 1-Intermediate (30 to 60 °)  2-Large (> 60 °) |
| OS* | Panicle shape | 1-Erect primary branches  2-Drooping primary branches  3-Elliptic  4-Semi-compact  5-Semi-open  6-Type Broom |
| PD* | Panicle density | 1-Very open  2-Open  3-Semi-open  4-Semi-compact  5-Compact |
| LPMR* | Length of the Panicle main rachis | 1-Short (3 to 6 cm)  2-Intermediate (6,1 to 12 cm)  3-Long (12,1 to 24cm)  4-Very long (> 24cm) |
| LPBP* | Length of the primary branch of the panicle | 1-Very short (<3cm)  2-Short (3 to 6 cm)  3-Intermediate (6,1 to 12 cm)  4-Long (12,1 to 24cm) |
| SEP* | Shape and extension of the peduncle | 1-Elongated (2a10cm)  2-Very elongated (> 10cm)  3-Recurvado (gooseneck) |
| LPF* | Length of the pedicellated flower | 1-Intermediate  2-Long  3-Very long |
| GC* | Glume color | 1-White  2-Grey  3-Yellow  4-Brown |
| PGA | Pigmentation of the glume by anthocyanin | 1-Null  2-Red  3-Purple |
| PGPA | Pigmentation of the glume pubescence by anthocyanin | 1-Null  2-Red  3-Purple |
| PLAL | Presence and length of the awn in the lemma | 1-Short  2-Intermediate  3-Long |
| FAP* | Formation of the awn in the palea | 1-Absent  2-Present |
| SP* | Stigma pigmentation | 1-Null  2-Yellow  3-Purple |
| OP* | Ovary pigmentation | 1-White  2-Purple |
| DAP | Dry anthers pigmentation | 1-White  2-Yellow  3-Green  4-Purple |
| DSP | Dry stamen pigmentation | 1-Light yellow  2-Orange  3-Red  4-Dark Red |
| GC1* | Grain covering | 1- 25% grain covered  2- 50% grain covered  3- 75% grain covered  4- Grain fully covered |
| GC2* | Grain color | 1-White  2-Grey  3-Cream  4-Yellow  5-Light Brown  6-Brown |
| DGF | Dorsal grain form | 1-Narrow elliptical  2-Elliptical  3-Circular |
| PGF* | Profile grain form | 1-Narrow elliptical  2-Elliptical  3-Circular |
| SW* | 1000-seed weight | 1-16 g  2-17 g  3-18 g  4->20 g |
| PFG | Presence of forehead on the grain | 1-Absent  2-Present |
| EC* | Endosperm composition | 1-75% amylopectin  2-Sweet |
| ET* | Endosperm texture | 1- Completely corneous  2- Mostly corneous  3- Intermediate  4- Mostly starchy  5- Completely starchy |
| EC1* | Endosperm color | 1-White  2-Yellow |
| GL* | Grain luster | 1- Lustrous  2- Not lustrous |
| PCP* | Purple color on the pericarp | 1-Absent  2-Present |
| TC1* | Threshing capacity | 1-1 a 5%  2-6 a 10% |

* Morphological descriptors used in the diversity analysis.
