# Supplementary material for: Phenotypic and molecular characterization of sweet sorghum accessions for bioenergy production
Source: PLoS One. 2017 Aug 17;12(8):e0183504. doi: 10.1371/journal.pone.0183504 (PMC5560702; doi:10.1371/journal.pone.0183504)
Supplement: S4 Table — Number of SNPs before and after data filtering for a minor allele frequency (MAF) of 5% and a maximum of 5% of missing genotypes per locus, final chromosome coverage and final marker density. (DOCX) [file pone.0183504.s004.docx]

**S4 Table. SNP markers used for molecular characterization.** Number of SNPs before and after data filtering for a minor allele frequency (MAF) of 5% and a maximum of 5% of missing genotypes per locus, final chromosome coverage and final marker density.

| **Chromosome** | **SNP markers^*^** | **SNP markers^**^** | **Chromosome**  **coverage(bp)^**^** | **SNP marker density (bp)^**^** |
| --- | --- | --- | --- | --- |
| **1** | 71,557 | 7,019 | 73,720,953 | 10,503 |
| **2** | 52,570 | 5,302 | 77,619,166 | 14,640 |
| **3** | 58,093 | 5,595 | 74,389,947 | 13,296 |
| **4** | 45,485 | 4,442 | 67,951,981 | 15,298 |
| **5** | 23,611 | 2,425 | 62,219,406 | 25,657 |
| **6** | 36,499 | 3,764 | 62,134,415 | 16,507 |
| **7** | 27,717 | 2,666 | 64,218,591 | 24,088 |
| **8** | 21,823 | 2,327 | 55,338,475 | 23,781 |
| **9** | 31,554 | 3,214 | 59,412,721 | 18,486 |
| **10** | 34,524 | 3,452 | 61,062,962 | 17,689 |
| **Total** | 403,433 | 40,206 | 658,068,617 | - |
| **Average** | 40,343 | 4,021 | 65,806,862 | 17,994 |

^*^before and ^**^ after filtering for a MAF of 5% and a maximum of 5% of missing genotypes per locus; bp: base pairs.
