# Supplementary material for: Phenotypic and molecular characterization of sweet sorghum accessions for bioenergy production
Source: PLoS One. 2017 Aug 17;12(8):e0183504. doi: 10.1371/journal.pone.0183504 (PMC5560702; doi:10.1371/journal.pone.0183504)
Supplement: S5 Table — (DOCX) [file pone.0183504.s005.docx]

**S5 Table.** Averages and standard deviations (SD) of the agro-industrial traits obtained for each cluster identified through the molecular and the phenotypic diversity analysis.

| **Cluster** | **CEL** | **EXT** | **FBY** | **FLOW** | **HEM** | **LIG** | **PH** | **POL** | **RSJ** | **TSS** |
| --- | --- | --- | --- | --- | --- | --- | --- | --- | --- | --- |
|  | **Molecular Diversity Analysis** | | | | | | | | | |
| **I-M** | 34.20 ± 2.21^c^ | 65.46 ± 4.92^a^ | 58.16 ± 15.21^a^ | 84.57 ± 6.19^a^ | 20.08 ± 1.74^c^ | 4.97 ± 0.74^d^ | 2.93 ± 0.26^ab^ | 10.28 ± 1.71^a^ | 1.36 ± 0.18^d^ | 15.38 ± 1.91^a^ |
| **II-M** | 37.35 ± 2.46^b^ | 66.03 ± 3.31^a^ | 51.40 ± 1.92^b^ | 79.92 ± 3.96^b^ | 21.79 ± 1.91^b^ | 5.98 ± 0.65^b^ | 3.04 ± 0.28^a^ | 9.77 ± 2.06^a^ | 1.62 ± 0.30^ab^ | 14.95 ± 2.19^a^ |
| **III-M** | 39.82 ± 3.10^a^ | 60.37 ± 6.64^c^ | 49.12 ± 12.57^b^ | 84.95 ± 6.98^a^ | 22.34 ± 1.69^ab^ | 6.31 ± 0.99^ab^ | 2.78 ± 0.51^b^ | 9.08 ± 2.21^b^ | 1.44 ± 0.25^cd^ | 13.99 ± 2.32^b^ |
| **IV-M** | 40.08 ± 2.90^a^ | 58.36 ± 7.52^c^ | 45.21 ± 12.58^c^ | 76.94 ± 5.94^c^ | 23.44 ± 1.91^a^ | 6.65 ± 0.97^a^ | 2.73 ± 0.40^b^ | 7.95 ± 2.29^c^ | 1.54 ± 0.32^bc^ | 12.71 ± 2.38^c^ |
| **V-M** | 37.35 ± 2.03^b^ | 64.82 ± 4.89^a^ | 31.02 ± 13.38^d^ | 72.94 ± 8.3^d^ | 22.77 ± 1.60^ab^ | 6.13 ± 0.63^b^ | 2.47 ± 0.42^c^ | 6.83 ± 1.96^d^ | 1.73 ± 0.30^a^ | 12.10 ± 1.85^c^ |
| **VI-M** | 35.26 ± 2.91^c^ | 63.06 ± 5.50^b^ | 46.26 ± 9.92^c^ | 79.76 ± 6.12^b^ | 21.99 ± 1.96^b^ | 5.50 ± 0.79^c^ | 2.48 ± 0.36^c^ | 8.30 ± 2.64^bc^ | 1.56 ± 0.36^bc^ | 14.30 ± 2.65^ab^ |
|  | **Phenotypic Diversity Analysis** | | | | | | | | | |
| **I-P** | 34.52 ± 2.66^d^ | 62.53 ± 4.49^a^ | 49.82 ± 15.18^a^ | 82.10 ± 7.35^a^ | 20.44 ± 1.94^b^ | 5.14 ± 0.85^c^ | 2.91 ± 0.29^a^ | 8.83 ± 2.04^a^ | 1.49 ± 0.24^b^ | 16.84 ± 2.17^a^ |
| **II-P** | 37.59 ± 3.41^b^ | 65.31 ± 4.44^a^ | 42.94 ± 12.36^b^ | 77.22 ± 5.52^b^ | 22.95 ± 2.16^a^ | 6.10 ± 0.99^ab^ | 2.65 ± 0.42^b^ | 6.62 ± 2.44^c^ | 1.77 ± 0.36^a^ | 11.78 ± 2.44^c^ |
| **III-P** | 39.63 ± 2.62^a^ | 57.57 ± 6.01^b^ | 44.74 ± 9.51^b^ | 76.51 ± 6.38^bc^ | 22.78 ± 1.43^a^ | 6.44 ±0.87^a^ | 2.80 ± 0.49^a^ | 9.45 ± 1.86^ab^ | 1.69 ± 0.21^a^ | 14.31 ± 2.01^b^ |
| **IV-P** | 38.12 ± 3.15^b^ | 58.58 ± 6.23^b^ | 51.85 ± 14.98^a^ | 76.03 ± 7.74^c^ | 22.06 ± 1.72^a^ | 6.23 ±0.91^a^ | 2.84 ± 0.42^a^ | 8.64 ± 1.96^b^ | 1.49 ± 0.24^b^ | 13.58 ± 2.10^b^ |
| **V-P** | 36.11 ± 2.66^c^ | 65.49 ± 5.67^a^ | 51.36 ± 11.16^a^ | 71.24 ± 6.16^d^ | 21.87 ± 1.57^a^ | 5.67 ± 0.79^b^ | 2.35 ± 0.33^c^ | 9.03 ± 1.76^a^ | 1.46 ± 0.22^b^ | 16.01 ± 1.79^a^ |

Averages within each type of diversity analysis exhibiting the same letter are not significantly different at $\propto=0.05$ according to Duncan´s test. CEL: cellulose (%); EXT: juice extraction (%); FBY: fresh biomass yield (t.ha^-1^); FLOW: days to flowering (in days after sowing); HEM: hemicellulose (%); LIG: lignin (%); PH: plant height (m); POL: sucrose concentration in juice (%); RSJ: reducing sugars in the juice (%) and TSS: total soluble solids (°Brix).
